# SnO_2_ ink engineering for printing efficient flexible perovskite solar modules

**DOI:** 10.1126/sciadv.adu1116

**Published:** 2025-10-24

**Authors:** Chao Wang, Yanping Mo, Xiaofeng Gao, Qinyang Huang, Tongle Bu, Qi Li, Yi-Bing Cheng, Fuzhi Huang

**Affiliations:** ^1^ Research Center for Advanced Thin Film Photovoltaics, State Key Laboratory of Advanced Technology for Materials Synthesis and Processing, Wuhan University of Technology, Wuhan 430070, P.R. China.; ^2^ National Energy Key Laboratory for New Hydrogen-Ammonia Energy Technologies, Foshan Xianhu Laboratory, Foshan 528200, P.R. China.

## Abstract

Flexible perovskite solar cells have broader prospects for application over their rigid counterparts. However, they are normally prepared by the spin coating process, which is not conducive to scaling up. One of the considerable barriers to scaling up stems from the printing of electron transport layers (ETLs), with tin oxide (SnO_2_) being a commonly used material. Here, poly(acrylic acid) (PAA) is introduced into the SnO_2_ nanocrystals ink to engineer the ink to enhance the dispersion of nanocrystals and slow down solvent evaporation, ensuring the printed ETLs having optimized coverage and morphology. Concurrently, the buried interface is refined by PAA, which enhances charge transfer and suppresses nonradiative recombination. The flexible device modified by PAA achieves a high efficiency of 22.46% (certified at 21.56%) and retains 89.3% of its initial value after 3000 bending cycles and 92.4% after 2000 hours of storage. The printed 30 centimeter–by–30 centimeter flexible module attains an impressive efficiency of 16.40% (certified at 16.28%).

## Teaser

Through the engineering of SnO_2_ ink, scalable printing of efficient and stable flexible perovskite solar modules is enabled.

## INTRODUCTION

Perovskite solar cells (PSCs) have seen rapid development and are progressively advancing toward industrialization. Flexible PSCs (F-PSCs) offer numerous advantages over their rigid counterparts, such as being thin, flexible, and having a broader spectrum of applications, including serving as a portable power source for flexible electronics (*1*–*4*). Despite notable efficiency breakthroughs in small-area F-PSCs (*5*–*8*), there is an apparent decrease in efficiency upon scaling up (*9*–*11*), which is considerably lower compared to the efficiency of large-area rigid modules (*12*–*15*). In contrast to inverted PSCs that rely heavily on high-energy vacuum processes (*16*), n-i-p structured PSCs are amenable to wet printing processes (*17*, *18*), suggesting a substantial potential for future cost reduction in mass production. Tin oxide (SnO_2_) nanocrystals (NCs) are extensively used for the electron transport layer (ETL) due to their high carrier mobility and cost-effectiveness. As the thinnest functional layer, the ETL is often the bottleneck in the fabrication of large-area flexible devices (*19*, *20*). Now, research is predominantly centered on the spin coating process, which is not amenable to scaling up (*21*, *22*). The lack of in-depth research on scalable technologies is a considerable barrier to the advancement of F-PSCs (*23*).

Bu *et al.* (*24*) prepared a flexible mini-module in a size of 5 cm by 6 cm by slot-die printing SnO_2_ electron transport layers (ETLs), achieving an efficiency of 15.22%. However, to ensure drying uniformity, hot air is used to assist in the drying process. Consequently, when attempting to scale up further, maintaining uniformity becomes challenging (*25*–*28*). Grapperhaus *et al.* (*29*, *30*) used acetic acid modified SnO_2_ dispersed in ethanol to blade-coat SnO_2_ films directly onto perovskite films, yielding flexible p-i-n small-area PSCs. Lee *et al.* (*11*) presented a large-area flexible perovskite solar module (F-PSM) with an area of 900 cm^2^, using a sequential blading coating of SnO_2_ NCs and nanorods, complemented by a spin-coated perovskite layer. Therefore, the primary challenge in printing large-area flexible n-i-p PSMs lies in achieving a uniform ETL over a large area, with the key being the regulation of SnO_2_ ink that is compatible with large-area slot-die printing. Unlike perovskite inks, which are true solutions without issues of homogeneity or sedimentation, SnO_2_ ink is a slurry with a very low solid content, prone to agglomeration and settling of NCs (*17*, *31*). Meanwhile, chelation, including small-molecule compound (*32*, *33*) and macromolecular polymers (*34*–*37*), can enhance the performance of SnO_2_ films prepared by spin coating. Notably, polymers have a greater number of functional groups compared to small molecules, enabling them to more effectively chelate SnO_2_ NCs to form network in ink to stabilize the ink and inhibit the NC agglomeration. Numerous functional groups, including amino (*38*), sulfonic (*39*), and carboxylic groups (*29*, *30*, *40*), can chelate with SnO_2_ NCs. Among them, carboxylic groups are more widely used due to their superior aqueous solubility and ability to form strong chelation bonds with indium tin oxide (ITO) substrates (*37*, *40*–*42*). This is particularly important for flexible substrates, whose inherently low surface energy renders them poorly wettable (*43*). Polymers with carboxylic groups improve substrate contact, enhance coating uniformity, and strengthen the interface robustness of flexible devices, thereby boosting their mechanical bending resistance. For the fabrication of large-area flexible modules via slot-die coating, the uniformity and stability of the ink are also of paramount importance. The ink used for spin coating and even for doctor blading (*44*) is not compatible with slot-die coating (*45*), because the kinetics of wet film and dry film formation are completely different. Unfortunately, there is a scarcity of research exploring inks specifically tailored for large-area slot-die coating of SnO_2_ ETL on flexible ITO substrates. Thus, the modification of SnO_2_ ink with carboxyl-rich polymers to regulate the coating and drying kinetics and thereby forming a dense film are decisive factors in determining the feasibility of large-scale printing (*36*, *46*, *47*).

Considering the aforementioned challenges, poly(acrylic acid) (PAA) is introduced into the ink to regulate the dispersion of SnO_2_ NC and optimize the wet film drying process during slot-die printing. Acting as a coordination and delay-drying agent, PAA facilitates the cross-linking of SnO_2_ NCs, mitigates the rapid volatilization of solvents, and prevents the irregular precipitation and agglomeration of NCs during natural drying. This approach ultimately leads to the formation of a smooth, uniformly distributed, and compact SnO_2_ ETLs, on which high-efficiency and bend-resistant F-PSCs are fabricated with a certified efficiency of 21.56%, retaining 89.3% of its initial value after 3000 bending cycles. The upscaled F-PSM in the size of 30 cm by 30 cm attains an efficiency exceeding 16% (certified at 16.28%).

## RESULTS

### Slot-die printed high-quality SnO_2_ films

An optimal concentration of multidentate chelating agent, PAA (0.5 mg/ml), having multiple carboxylic groups (molecular formula depicted in fig. S1), was introduced into the SnO_2_ NC ink to render it suitable for slot-die printing large-area SnO_2_ films. Both the control ink and the PAA-modified ink were applied to a 30 cm–by–30 cm polyethylene naphthalate (PEN)/ITO flexible substrate via slot-die printing. The entire preparation process occurred at ambient temperature without the assistance of gas blowing. The resulting films are designated as conventional SnO_2_ (C-SnO_2_) and PAA-modified SnO_2_ (P-SnO_2_), respectively.

**Fig. 1.**
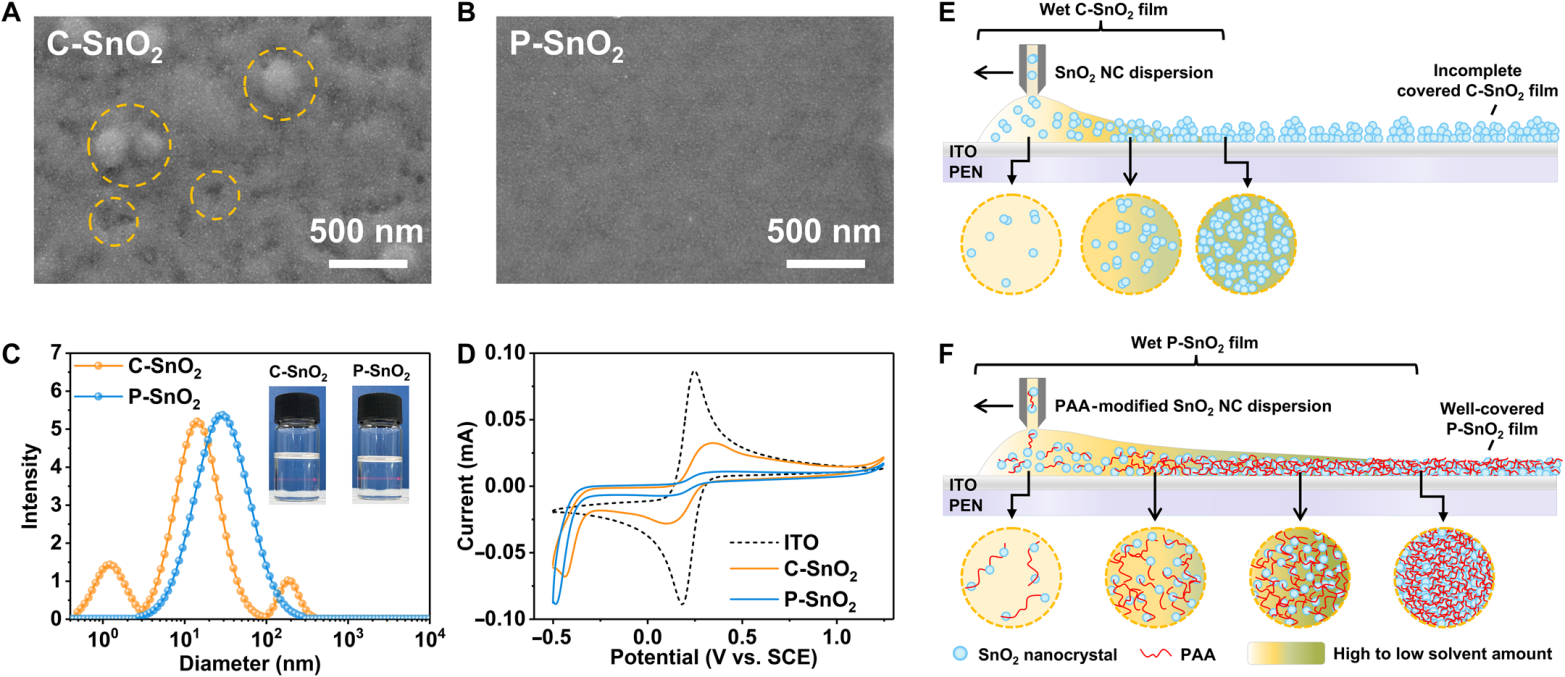
The performance of P-SnO_2_ NC inks and films. The top view SEM morphology of (**A**) the C-SnO_2_ and (**B**) P-SnO_2_ films. (**C**) The DLS plots of the C-SnO_2_ and P-SnO_2_ inks, and the inset displays their digital photos. (**D**) The CV plots of the C-SnO_2_ and P-SnO_2_ films. The schematic diagrams of slot-die printing process of (**E**) the C-SnO_2_ and (**F**) P-SnO_2_ films.

The morphology of the SnO_2_ films was examined using scanning electron microscopy (SEM). As illustrated in Fig. 1A, the SEM image of the C-SnO_2_ film reveals obvious large particle protuberances and voids, which hinder efficient charge transfer between the perovskite and the SnO_2_ ETL. Moreover, with the assistance of an N_2_ knife for drying, the number of surface particles was reduced, but the formation of pores could not be completely inhibited (fig. S2A). In contrast, the P-SnO_2_ film with PAA of a molecular weight (MW) of ~450,000, as shown in Fig. 1B, exhibits a smooth and even surface regardless of whether an N_2_ knife is used. PAA with an MW of ~2000 is insufficient to eliminate pinholes in SnO_2_ films, whereas PAA with an MW of ~4,000,000 is poorly soluble and incompatible with slot-die coating processes (figs. S3 and S4). The shear viscosity (shear rate = 100 s^−1^) of C-SnO_2_ ink is close to that of P-SnO_2_ ink with an MW of ~2000 PAA. The MW of PAA correlates positively with ink viscosity: Longer chains are more likely to form interwoven three-dimensional (3D) networks, thereby reducing the fluidity of the inks (fig. S5). Furthermore, the increased viscosity decreases the Marangoni flow in wet films, which in turn suppresses the coffee ring effect (*48*). The Marangoni effect was investigated by drop-casting different inks onto substrates and allowing them to dry naturally (fig. S6). The C-SnO_2_ and P-SnO_2_ (MW, ~2000) films exhibit a pronounced coffee ring effect, whereas the P-SnO_2_ film (MW, ~450,000) shows a more uniform distribution. This ultimately leads to the formation of a denser and more even P-SnO_2_ film. Hereafter, ink modified with PAA of an MW ~450,000 is used as the target sample.

To delve into the mechanism by which PAA influences the quality of SnO_2_ films, further analysis of the ink was conducted. The particle size distribution of the NCs was ascertained through dynamic light scattering (DLS). As depicted in Fig. 1C, the C-SnO_2_ ink exhibits NC size distributions at ~2, 15, and 200 nm, respectively. In contrast, the size distribution of NCs in the PAA-modified ink is concentrated around 30 nm, suggesting that PAA-induced self-aggregation yields slightly larger, monodisperse colloidal aggregates (*36*, *47*). Slightly larger colloidal aggregates indicate increased cross-linking without altering individual NC dimensions. During the drying process, as the solvent evaporates, the aggregates are subjected to capillary forces between the particles, which generate a contraction effect. This further collapses the aggregates, leading to a reduction in film thickness and increased film coverage. Furthermore, the Tyndall effect of P-SnO_2_ ink is marginally more pronounced than that of the C-SnO_2_ ink, indicating increased NC diameter in the P-SnO_2_ ink.

The quantity of PAA influences the properties of the SnO_2_ ink. When the concentration of PAA surpasses 1 mg/ml, a portion of the ink starts to form a gel-like sediment (fig. S7A). With the concentration of PAA reaches 2 mg/ml, it can even transform into an opaque, viscous gel (fig. S7B). Given that a high concentration of PAA (5 mg/ml) remains clear and transparent when dissolved in water (fig. S7C), these observations imply that PAA alters the surface characteristics of the colloidal particles. Simultaneously, the viscosity also increases rapidly with the addition amount of PAA (fig. S8). Notably, the absolute value of the zeta potential of C-SnO_2_ ink (−24.82 mV) is lower than that of P-SnO_2_ ink [−40.7 mV for PAA (0.5 mg/ml) and −49.07 mV for PAA (1 mg/ml)], indicating that the PAA-modified inks have a better stability (fig. S9 and table S1) (*47*). After 4 months of storage, the diameter of NCs in C-SnO_2_ ink exhibits an apparent increase, whereas the P-SnO_2_ ink maintains similar diameter distribution with that of the fresh ink (fig. S10). The robust chelation between PAA and SnO_2_ NCs in the ink strengthens the interparticle forces among P-SnO_2_ NCs, subsequently modifying the dispersion of the SnO_2_ NCs in the ink. Cyclic voltammetry (CV) measurements indicate that the P-SnO_2_ film provides superior coverage over the control sample (Fig. 1D), with no evident leakage current detected (*49*). Furthermore, ultraviolet-visible (UV-vis) spectroscopy analysis of the film transmittance reveals that the P-SnO_2_ film has higher transmittance (fig. S11), attributed to its smooth surface morphology.

A comparison is then made of the effects of different inks on the wet film during the printing process. As shown in fig. S12, through real-time monitoring of the mass change of the wet films by slot-die coating different SnO_2_ inks, it is observed that the solvent evaporation rate in the wet film prepared with C-SnO_2_ ink is apparently faster. The film dries completely in ~4 s, whereas the P-SnO_2_ film requires around 10 s to dry. Figure 1 (E and F) illustrates the drying process of the SnO_2_ wet film during slot-die printing. As water evaporates, the PAA segments interlock more tightly, increasing in concentration until the once fluid wet film transitions into a viscous state, which slows the drying rate of the wet film. During this drying phase, the fluidity of the wet film is sharply curtailed, attenuating surface tension–driven convection, thereby suppressing the Marangoni effect. The reduction of liquid flow rate further enhances the uniformity of the film and preventing uneven drying areas that could result from irregular ink flow. Concurrently, the gaps between P-SnO_2_ NCs in the wet film gradually diminish under the tensile effect of PAA, culminating in the formation of a dense and even P-SnO_2_ film.

### Enhanced buried interface characteristics

**Fig. 2.**
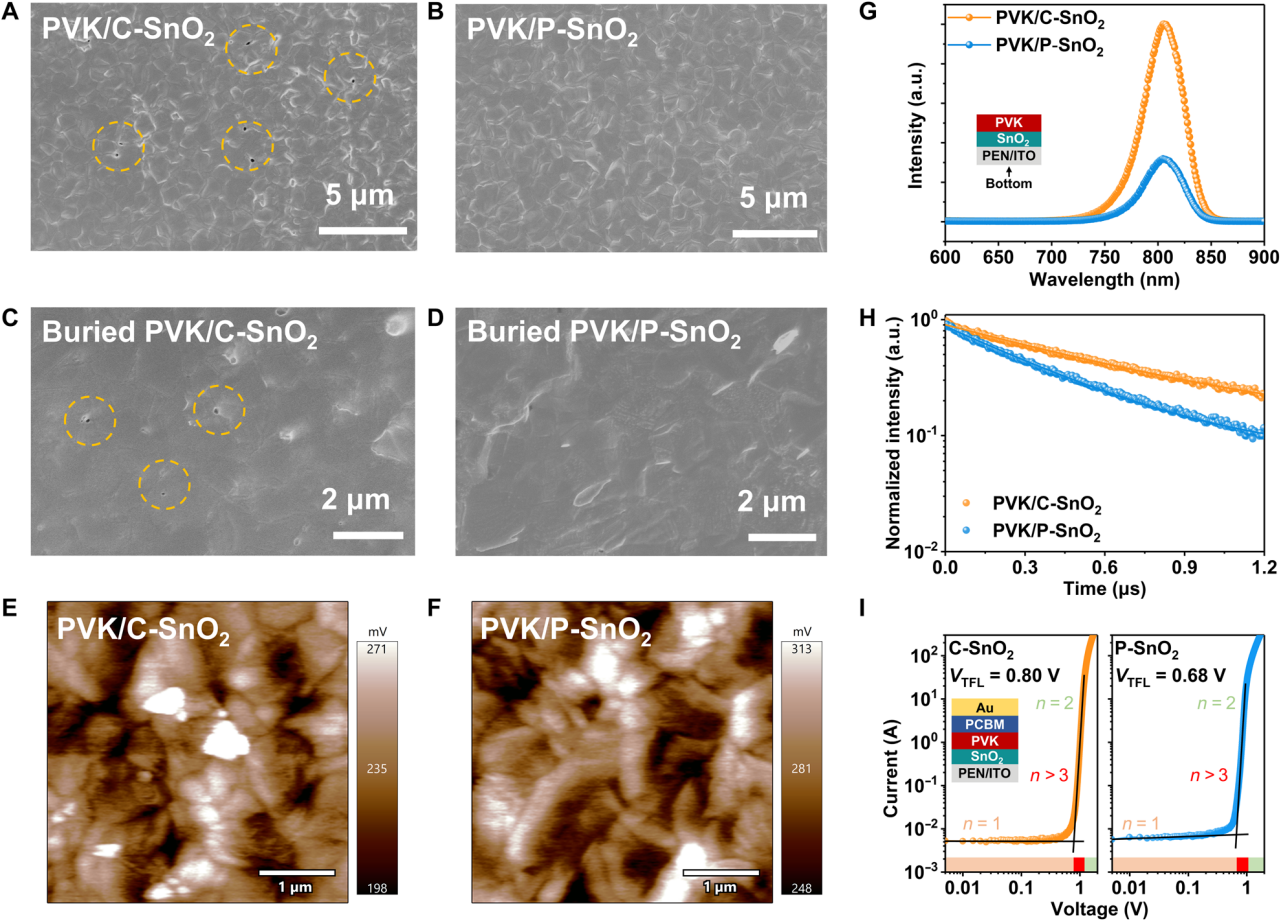
Properties of the perovskite films deposited on different SnO_2_ films. The top view SEM morphology of perovskite (PVK) films fabricated on the (**A**) C-SnO_2_ and (**B**) P-SnO_2_. The SEM morphology of buried interface of (**C**) PVK/C-SnO_2_ and (**D**) PVK/P-SnO_2_. The Kelvin probe force microscopy surface potential images of PVK films deposited on (**E**) PVK/C-SnO_2_ and (**F**) PVK/P-SnO_2_. (**G**) Steady-state PL spectra and (**H**) TRPL decays of PVK films on different SnO_2_ films. (**I**) Dark *I*-*V* curves of the electron-only devices, the detailed structure is showed in inset. a.u., arbitrary units.

Perovskite films were deposited on the slot-die printed SnO_2_ ETLs. A comparison of the morphology on the upper surface of the perovskite films reveals that the perovskite film on C-SnO_2_ exhibits noticeable pinholes, whereas the film on P-SnO_2_ is dense and devoid of holes (Fig. 2, A and B). The characteristics of the ETL surface profoundly influence the buried interface, a critical aspect within the device structure (*50*, *51*). By applying a UV-curable adhesive to the surface of the perovskite film, it can be detached from the flexible substrate (*10*). The buried interface of C-SnO_2_/perovskite shows some holes, but the interface of P-SnO_2_/perovskite maintains a dense morphology, free of pinholes (Fig. 2, C and D). Transmission optical microscopy (fig. S13) indicates that the perovskite films on C-SnO_2_ have an irregular light and shade distribution, suggesting a nonuniform thickness of the perovskite layer. This is detrimental to efficient light harvesting, leading to a loss in photocurrent. The atomic force microscope image in fig. S14 reveals that the average surface roughness of the perovskite film on P-SnO_2_ (*R*a = 30.062) is lower than that of the film on C-SnO_2_ (*R*a = 37.366). These morphological differences suggest that PAA enhances interfacial contact and improves the quality of the perovskite films.

Besides the uniformity of the ETLs, the wettability and the surface groups of the ETLs obviously influence the characteristics of the perovskite layer. To assess the wettability of various ETLs, contact angle measurements were conducted (fig. S15). The contact angle between the perovskite precursor solution and P-SnO_2_ is ~45°, which is notably lower than the angle observed with C-SnO_2_, at around 65°. The superior wettability of P-SnO_2_ facilitates effective wetting during the printing process, leading to the formation of a uniform and compact perovskite film (*10*, *52*). Fourier transform infrared (FTIR) spectroscopy was used to analyze the surface groups of the ETLs. As shown in fig. S16, the intensities of the characteristic peaks in the reflectance mode FTIR spectra of C-SnO_2_ and P-SnO_2_ are very close, suggesting that the amount of PAA distributed on the P-SnO_2_ surface is very low. Further analysis of the surface groups on ETLs was conducted using x-ray photoelectron spectroscopy (XPS). The K 2p peak intensity for both ETLs is found to be nearly identical, as shown in fig. S17 and table S2. However, a slight increase in the peak intensity is observed for C─C (284.8 eV) and O─C═O (288.8 eV) in the C 1s spectrum of P-SnO_2_, while the peak intensity for C─O (286.6 eV) remains largely unchanged. The slight enhancement of the characteristic peak of the carboxyl group suggests that the PAA is distributed on the film surface (*40*). The increased carboxyl group content enhances wettability. X-ray diffraction (XRD) patterns shows that the full width at half maximum of the (100) diffraction peak for the perovskite on P-SnO_2_ narrows with the increase in intensity. This indicates that the incorporation of PAA also enhances the crystallinity of the perovskite, as shown in fig. S18.

The energy levels of SnO_2_ ELTs were characterized using ultraviolet photoelectron spectroscopy (UPS) and UV-vis spectroscopy. It is found that the conduction band minimum (CBM) of the P-SnO_2_ film is more closely aligned with that of the perovskite film, as shown in fig. S19 and detailed in table S3. To further assess the potential of perovskite on various ETLs, Kelvin probe force microscopy was used (Fig. 2, E and F). The data indicate that the average potential of perovskite on P-SnO_2_ is higher, which corresponds to a lower work function, suggesting that charge recombination at the P-SnO_2_ and perovskite interface is suppressed, while the charge transfer rate is enhanced (*53*). The well-matched band structure facilitates faster charge transfer and reduces interface recombination, thereby optimizing the performance of the F-PSCs.

Steady-state photoluminescence (PL) and time-resolved PL (TRPL) spectroscopies were used to delve into the charge transfer and recombination dynamics of perovskite films in the ITO/SnO_2_/perovskite structure. As shown in Fig. 2G, the PL peak intensity for the perovskite film on P-SnO_2_ is markedly lower compared to the C-SnO_2_ sample, suggesting that P-SnO_2_ facilitates more rapid electron extraction than C-SnO_2_. Figure 2H presents the TRPL decay of the perovskite on P-SnO_2_, which was fitted with a single exponential function (table S4). The time constant (τ) for the perovskite on P-SnO_2_ is determined to be 393 ns, shorter than that of C-SnO_2_ at 641 ns, indicating that the presence of PAA apparently suppresses nonradiative recombination at the buried interface (*54*). As shown in fig. S20, the TRPL mapping image of the perovskite film based on C-SnO_2_ exhibits some dark areas, corresponding to perovskite pinholes due to poor contact at the bottom interface. In contrast, the perovskite film based on P-SnO_2_ shows a relatively uniform lifetime distribution, indicating that the perovskite layer is uniformly distributed on the P-SnO_2_ surface. Conductivity measurements on the ITO/SnO_2_/Au structure (fig. S21) reveal that the introduction of insulating PAA leads to slightly reduced conductivity in P-SnO_2_. The slight reduction in conductivity hampers carrier transport but improves coverage, and planarity in turn enhances the overall interface extraction rate. The dark current-voltage relation (*I*-*V*) characteristics of ITO/SnO_2_/FAPbI_3_/[6,6]-phenyl-C_61_-butyric acid methyl ester (PCBM)/Au devices, as shown in Fig. 2I, were used to assess the interface defect density. The calculated trap filling limit voltage (*V*_TFL_) is reduced from 0.80 to 0.68 V, and the defect state density (*N*_t_) is also lowered from 1.38 × 10^16^ to 1.17 × 10^16^ cm^−3^. This reduction clearly indicates that the P-SnO_2_–based device has a lower defect density at the buried interface, attributed to the denser, flatter nature of the P-SnO_2_ film and its better energy level alignment (*55*).

### Device performance and characterization

**Fig. 3.**
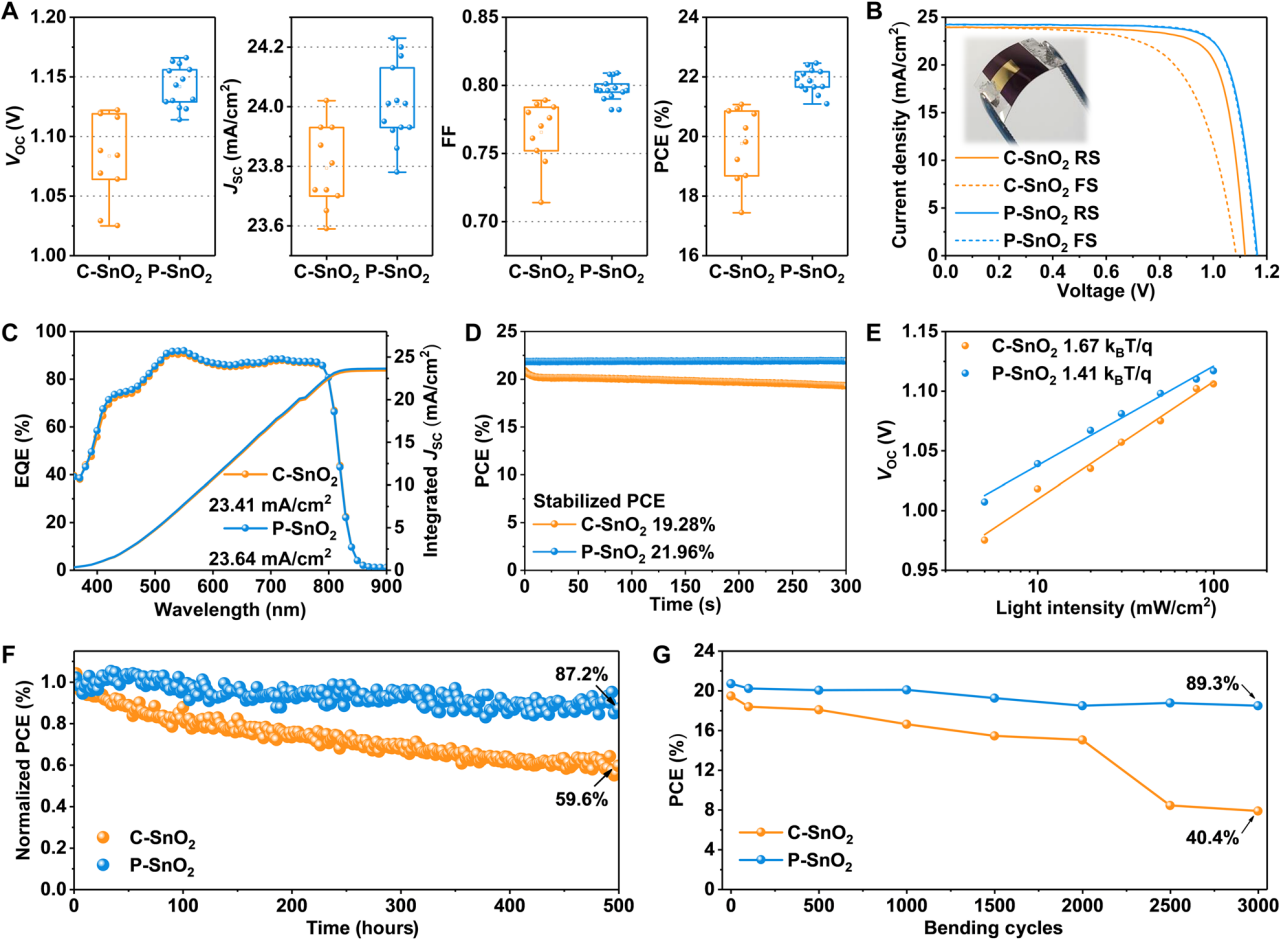
Device performance and characterization. (**A**) Performance comparison of the F-PSCs based on the C-SnO_2_ and P-SnO_2_ ETLs. Device counts are 10 for each type. The top and bottom horizontal lines of the box plots denote the 75th percentile and the 25th percentile, respectively. The middle point represents the average value. The whiskers correspond to an outlier detection coefficient of 1.5. (**B**) The *J*-*V* curves, (**C**) external quantum efficiency (EQE) spectra, (**D**) steady-state power output, and (**E**) light-dependent *V*_OC_ of the best-performing F-PSCs based on the C-SnO_2_ and P-SnO_2_ ETLs. (**F**) ISOS-L-1 stability test of the FPSCs under MPPT conditions. All devices are encapsulated in advance and then tested in the atmospheric environment (60 ± 10% RH and 25° ± 5°C). (**G**) The bending durability (*R* = 5 mm, ~1 Hz, tension only) of unencapsulated F-PSCs under the ambient condition (~20% RH, ~25°C). FS, forward scanning.

To explore the performance of devices using various ETLs, F-PSCs with an active area of 0.1486 cm^2^ in configuration of ITO/SnO_2_/FAPbI_3_/i-butylammonium iodide (iBAI)/2,2′,7,7′-Tetrakis-(N,N-di-4-methoxyphenylamino)-9,9′-spirobifluorene (spiro-OMeTAD)/Au were fabricated. Before testing, all devices were aged in air at room temperature for a week. Figure 3A and fig. S22 present the histogram of device performance. The efficiencies of P-SnO_2_ films with thicknesses of about 22, 41, 76, and 150 nm measured by ellipsometry were 20.91, 21.99, 19.69, and 18.4%, respectively. As the thickness of P-SnO_2_ increases, the photostability first increases and then decreases (fig. S23). Thus, an optimized P-SnO_2_ layer with a thickness of ~41 nm was obtained. The variation in open-circuit photovoltage (*V*_OC_) and fill factor (FF) for devices based on C-SnO_2_ is broader compared to thickness-optimized P-SnO_2_ devices, a discrepancy attributed to the inferior uniformity of C-SnO_2_ films. In contrast, P-SnO_2_–based devices demonstrate superior efficiency, which can be attributed to their enhanced film density, improved interface contact, and more precise energy level alignment.

Figure 3B presents the current density-voltage (*J*-*V*) characteristics of the best-performing F-PSCs based on C-SnO_2_ and P-SnO_2_, as detailed in table S5. The P-SnO_2_–based device records a remarkable power conversion efficiency (PCE) of 22.54%, with a certified efficiency of 21.56% (fig. S24), which is substantially higher than that of the C-SnO_2_–based device at 21.07%. The P-SnO_2_–based devices exhibit superior *V*_OC_, short-circuit photocurrent (*J*_SC_), and FF. External quantum efficiency measurements, as shown in Fig. 3C, reveal that the P-SnO_2_–based device has higher values across all wavelengths, resulting in a higher generated current of 23.64 mA/cm^2^ compared to the C-SnO_2_–based device (23.41 mA/cm^2^). Under steady-state power output conditions at 300 s, the C-SnO_2_–based device experiences distinct degradation, whereas the P-SnO_2_–based device maintains its performance, with final efficiencies recorded at 19.28 and 21.96%, respectively, as shown in Fig. 3D.

Figure 3E illustrates the linear relationship between *V*_OC_ and light intensity, which is crucial for analyzing the charge recombination dynamics under open-circuit conditions. The slope of the P-SnO_2_–based devices, at 1.41 k_B_T/q, is notably lower than that of the C-SnO_2_–based devices, which is 1.67 k_B_T/q. This difference suggests that the P-SnO_2_–based devices are more effective at suppressing trap-assisted Shockley-Read-Hall recombination (*56*). The Mott-Schottky plots in fig. S25 provide further insight into the complete devices. It reveals that the built-in potential (*V*_bi_) for the P-SnO_2_–based device is 1.03 V, whereas for the C-SnO_2_–based device, it is 0.94 V. These values align with the *V*_OC_ trends observed during the *J*-*V* tests, underscoring the superior performance of the P-SnO_2_–based devices (*24*). In addition, the electrochemical impedance spectroscopy (EIS) curve shown in fig. S26 indicates that the recombination resistance of the P-SnO_2_ film is evidently higher at 1486 ohm compared to that of the C-SnO_2_ film (1082 ohm). This enhancement in resistance implies an acceleration in electron transmission at the buried interface and a corresponding decrease in nonradiative recombination (*47*).

The devices were stored at room temperature and 20% relative humidity (RH) for 2000 hours to evaluate their storage stability. As shown in fig. S27, the P-SnO_2_–based device retains an impressive 92.4% of its initial efficiency, whereas the C-SnO_2_–based device experiences a decline to just 67.0% of its initial performance. The maximum power point tracking (MPPT) test, conducted according to the International Summit on Organic Photovoltaic Stability (ISOS) protocol ISOS-L-1 standard, was to detect the operational stability of the devices. All devices were preencapsulated and then tested in an atmospheric environment (RH of 60 ± 10% and temperature of 25° ± 5°C) with a calibrated light intensity of 100 mW/cm^2^. The results shown in Fig. 3F indicate that the C-SnO_2_ device retains 87.2% of the initial efficiency after 500 hours of MPPT, while the P-SnO_2_ device retains 59.6% of its initial efficiency. Furthermore, the P-SnO_2_ device demonstrates remarkable flexibility, retaining 89.3% of its initial PCE after undergoing 3000 bending cycles, whereas the C-SnO_2_ device shows an apparent degradation, retaining only 40.4% of its initial value (Fig. 3G). The *J*_SC_ and FF of the C-SnO_2_ devices diminish markedly during the bending tests (fig. S28). In contrast, the P-SnO_2_ devices exhibit a slower rate of performance degradation and superior resistance to bending. This enhanced durability can be attributed to the incorporation of PAA, which refines the morphology, density, and surface adhesion of the ETL. Consequently, this leads to an increased charge transfer rate and a stronger contact force at the SnO_2_/perovskite interface, resulting in improved overall stability and resistance to mechanical stress.

### Scaling up

**Fig. 4.**
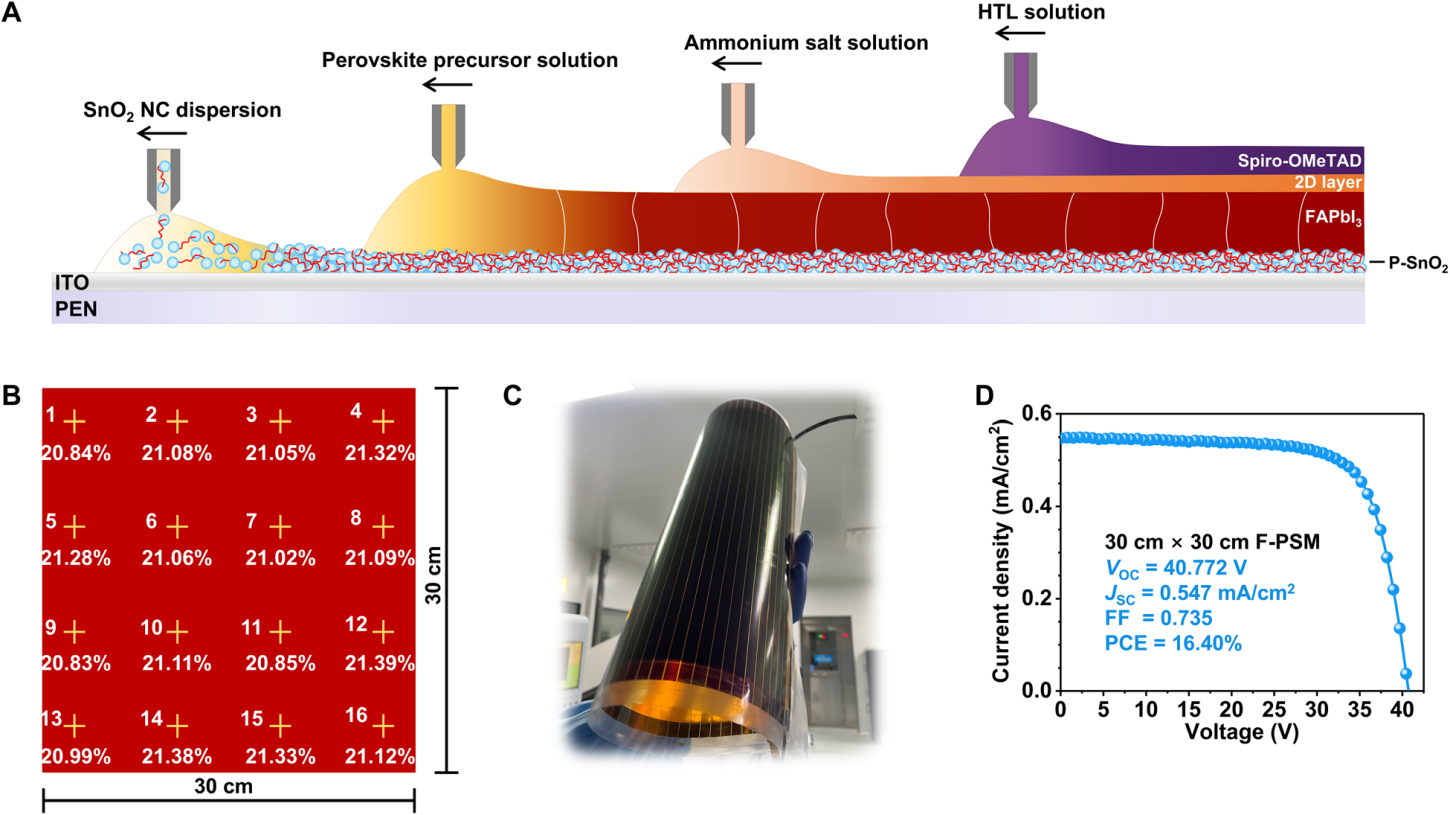
Performance of F-PSMs. (**A**) Schematic diagram of functional layers prepared by slot-die, including SnO_2_, PVK, 2D, and spiro-OMeTAD. (**B**) Efficiency distribution of small-area P-SnO_2_-based F-PSCs cut from 30 cm–by–30 cm printed films. (**C**) Photo of the encapsulated 30 cm–by–30 cm F-PSM. (**D**) The RS scan *J-V* curve of the champion P-SnO_2_-based F-PSM with an aperture area of 600 cm^2^.

To evaluate the feasibility of scale-up, the SnO_2_, perovskite, 2D, and spiro-OMeTAD layers were prepared by slot-die, as shown in Fig. 4A. The uniformity of the upscaled SnO_2_ films was evaluated by measuring thickness and transmittance across different regions. The results show that P-SnO_2_ films exhibit superior homogeneity compared to C-SnO_2_ (figs. S29 and S30). To check the uniformity of all functional layers by slot-die, the films were cut into small pieces according to the corresponding 16 regions, and gold electrodes were evaporated onto them. The photovoltaic characteristics distribution of the small devices is illustrated in Fig. 4B, fig. S31, and table S6. The efficiency distribution of P-SnO_2_ devices across different regions is consistent and exceeds 20.5%, whereas the efficiency distribution of C-SnO_2_ devices is irregular and generally lower.

Furthermore, a large-area F-PSM comprising 39 cells over 30 cm by 30 cm was fabricated, as shown in Fig. 4C and fig. S32. The widths of P1, P2, and P3 for laser processing of the dead area are detailed in fig. S33A, and the geometric FF is 92.3%. As demonstrated in Fig. 4D, the performance of the F-PSM is evaluated using a mask with an aperture area of 600 cm^2^ (fig. S33B). The P-SnO_2_ F-PSM achieves an efficiency of 16.40% (*J*_SC_ = 0.547 mA/cm^2^, *V*_OC_ = 40.772 V, FF = 0.735), with a certified efficiency of 16.28% (fig. S34). In contrast, the C-SnO_2_ F-PSM exhibits an efficiency of only 11.65% (fig. S35). The incorporation of PAA evidently enhances the uniformity of the SnO_2_ ETLs, perovskite layers, and the interfacial adhesion, which is pivotal for improving the efficiency of the F-PSMs.

## DISCUSSION

As the first layer of n-i-p F-PSCs, the challenge of scaling ETLs poses a critical barrier to the scaling up of F-PSCs. The SnO_2_ NCs, typically used for ETL coatings, are susceptible to aggregation and sedimentation in the ink, resulting in uneven film deposition and the introduction of numerous defects at the buried interface. Consequently, current research on F-PSCs mainly focuses on small-area devices, with spin coating being the primary processing method. To address this situation, we have carefully analyzed the SnO_2_ NC ink and introduced PAA as a dispersing agent to improve the quality of the ink. PAA coordinates the SnO_2_ NCs with multiple carboxylic groups, preventing them aggregation and stabilizing the ink. Using this refined ink for printing large-area SnO_2_ ETLs achieves a high-quality film that is uniform, dense, and smooth. This further improves the buried interface, promotes the subsequent perovskite growth, and then yields a certified efficiency of 16.28% for a 30 cm–by–30 cm F-PSM. This strategy improves the buried interface and strengthens adhesion between grains and substrate, increasing the bending resistance and providing a viable approach for the future industrial development of F-PSCs.

## MATERIALS AND METHODS

### Materials

Tin oxide colloidal dispersion (SnO_2_, 15% in H_2_O) was purchased from Alfa-Aesar. PAA (average MW, ~450,000), PAA (average MW, ~4,000,000), dimethylformamide (DMF), dimethyl sulfoxide (DMSO), ethyl acetate (EA), isopropyl alcohol (IPA), acetonitrile, tetrahydrofuran (THF), chlorobenzene, bis(trifluoromethane)sulfonimide lithium salt (Li-TFSI), 4-tert-butylpyridine (tBP), and tris(2-(1H-pyrazol-1-yl)-4-tert-butylpyridine)cobalt (III) tris(bis(trifluoromethylsulfonyl)imide)) (FK209) were purchased from Sigma-Aldrich. PAA (63% solids in H_2_O; average MW, ~2000) and potassium bis(fluoromethanesulfonyl)imide (K-FSI) were purchased from Aladdin. Methylammonium chloride (MACl), formamidinium iodide (FAI), lead iodide (PbI_2_), iBAI, and spiro-OMeTAD were purchased from Xi’an Polymer. Deionized water was self-made in the laboratory.

### Precursor solution preparation

#### Preparation of the C-SnO_2_ ink

The original SnO_2_ colloidal dispersion was diluted with deionized water at the volume ratio of 1:6 and centrifuged at 10,000 rpm for 5 min, and then the supernatant was taken and refrigerated for use.

#### Preparation of P-SnO_2_ ink

Different amounts of PAA with three kinds of MW are dissolved in an aqueous solution in advance, heated to 90°C, and stirred to dissolve it completely. The original SnO_2_ colloidal dispersion was diluted with cooled PAA solution at the volume ratio of 1:6 and then centrifuged at 10,000 rpm for 5 min. The mixture dispersion was stirred thoroughly at 4°C for a day, and then the supernatant was taken and refrigerated for use.

#### Preparation of perovskite precursor solution

The mixed perovskite precursor was prepared by dissolving 1.53 M PbI_2_ and 1.4 M FAI and 0.5 M MACl in the mixture solvent of DMF/DMSO (8:1, by volume).

#### Preparation of 2D solution

Two milligrams of iBAI and 2.8 mg of K-FSI were dissolved in 1 ml of mixture solvent of IPA/THF (95:5, by volume).

#### Preparation of spiro-OMeTAD solution

The spiro-OMeTAD solution was prepared by dissolving 91 mg of spiro-OMeTAD in 1 ml of chlorobenzene. Then, 23.5 μl of Li-TFSI [predissolved as a stock solution (520 mg/ml) in acetonitrile], 11 μl of FK209 [predissolved as a stock solution (300 mg/ml) in acetonitrile], and 36.5 μl of tBP were added.

### F-PSC and module preparation

#### Flexible ITO PEN substrate cleaning

The 30 cm–by–30 cm flexible substrates was etched with a femtosecond laser. Then, flexible substrates were sonicated in IPA for about 15 min. They were dried by nitrogen flow and ready for use.

#### C-SnO_2_ and P-SnO_2_ film deposition

The SnO_2_ films was slot-die coated with the coating speed of 0.5 m/min and the feeding rate of 0.25 ml/min, followed by annealing at 150°C for 30 min, and the gap was 120 μm.

#### Fabrication of F-PSCs

The 2 cm–by–2 cm substrates for F-PSCs were cut from 30 cm–by–30 cm flexible substrates with SnO_2_. Thirty microliters of the perovskite precursor solution was dropped and spread on the SnO_2_/ITO/PEN substrate. The substrate was then spun at 6000 rpm for 30 s with an accelerated speed of 2000 rpm. One hundred microliters of antisolvents of EA was dropped at the last 10th second. Then, the wet film was annealed at 100°C for 60 min in an N_2_ atmosphere. After the substrate cooling down, 30 μl of iBAI/K-FSI solution was deposited on the FAPbI_3_ perovskite layer by dynamic spin coating at 5000 rpm, followed by annealing at 100°C for 10 min. Then, 50 μl of spiro-OMeTAD solution was spun on the perovskite films at 3000 rpm for 30 s. The prepared films were placed in a drying cabinet (below 20% RH) for 12 hours to ensure the chlorobenzene completely volatilize. The gold layer was deposited in a two-step process: The first 5 nm at 0.1 Å/s and then at 3 Å/s up to 100 nm.

#### Fabrication of 30 cm–by–30 cm flexible modules

Three layers including perovskite layer, 2D layer, and hole transport layer (HTL) were slot-die coated on the SnO_2_/ITO/PEN substrates orderly. The coating process was carried out in ambient air. First, the wet film of perovskite precursor solution was applied on top of the UVO-treated SnO_2_/ITO/PEN substrate at the coating speed of 0.6 m/min and the feeding rate of 1.8 ml/min. In addition, the gap between the slot-die head and the substrate was 120 μm. The wet film was then dried by rapidly vacuuming to 5 Pa and holding for 10 s (*18*, *57*). The intermediate films were transferred onto a hotplate and annealed at 100°C for 60 min. The 2D layer was slot-die coated with the coating speed of 0.3 m/min and the feeding rate of 0.4 ml/min, followed by annealing at 100°C for 10 min, and the gap was 120 μm. The HTL was slot-die coated with the coating speed of 0.5 m/min and the feeding rate of 1 ml/min, and the gap was 180 μm. The prepared films were placed in a drying cabinet (below 20% RH) for 12 hours to ensure the chlorobenzene completely volatilize. The gold layer is deposited in a two-step process: the first 5 nm at 0.1 Å/s and then at 3 Å/s up to 100 nm. Note that all slot-die processes are prepared in ambient air (~20% RH, ~25°C).

#### Modularization

A femtosecond laser (FemtoYL, 1030 nm) was used to scribe P1 lines. A picosecond laser (GS-PGN30) was used to scribe P2 and P3 lines. The etching of P1 is completed before the PEN/ITO substrate is washed. P2 is etched after the HTL preparation. P3 is scribed after the Au electrode deposition. The schematic diagram of the scribing lines is shown in fig. S33A.

### Characterizations

#### SEM measurement

The SEM morphology of perovskite films was characterized by Hitachi S4800.

#### DLS and zeta potential measurement

DLS and zeta potential measurement was performed using a spectrophotometer (NanoBrook 90Plus PALS).

#### Rotary rheometer measurement

Shear viscosity of inks was performed using a rotary rheometer (Kinexus Pro+). The room temperature rheological energy cone-plate model was used for testing.

#### CV test

The CV test of SnO_2_ was investigated by electrochemical workstation (CHI660C Instruments, China) with a standard three-electrode system in 0.5 mM K_4_Fe(CN)_6_ + 0.5 mM K_3_Fe(CN)_6_ in 0.5 M aqueous KCl solution, where a saturated Hg, a Pt plate, and ITO/PEN substrate deposited with SnO_2_ worked as the reference, counter electrode, and working electrode respectively (*58*).

#### High-resolution XPS measurement

XPS was performed in 10^−10^ mbar ultrahigh vacuum with equipment Thermo Fisher Scientific ESCALAB 250Xi. The x-ray spot size was 500 μm, and the energy resolution was 50 meV. The monochromatic Al Kα (*h*ν = 1486.6 eV, *P* = 150 W) was used for XPS measurement. The C 1s spectrum was used to calibrate all XPS spectra, using 284.8 eV as the standard.

#### Energy level characterization

UPS measurement was performed with equipment Thermo Fisher Scientific ESCALAB 250Xi. He (*h*ν = 21.22 eV) was used as the excitation source for UPS measurement. UV-vis spectroscopy measurement was used to determine the energy band (*E*_g_). The work function (*W*_F_) and valence band binding energy were calculated from the secondary electron cutoff (*W*_F_ = 21.22 − *E*_cutoff_ − *E*_onset_).

#### XRD measurement

XRD (D8 Advance) with Cu Kα radiation (λ = 1.5406 Å) was performed to understand the crystal structure.

#### Space charge–limited current measurement

To quantitatively calculate the density of defects, we fabricate electron-only devices with a structure of ITO/SnO_2_/perovskite/PCBM/Au. At a low bias voltage, the linear *J*-*V* curve is the ohmic region. At an intermediate bias voltage, a trap-filled region is reached at a voltage (*V*_TFL_) where the current shows a rapid nonlinear rise and transfers into a trap-filled limit (TFL). During this regime, all the trap states were filled by the injected carriers. The relationship between trap density and the TFL regime is expressed as

$$n_{\text{trap}}=\frac{2V_{\text{TFL}}\varepsilon \varepsilon_{0}}{eL^{2}}$$

where *V*_TFL_ is the TFL voltage, ε is the relative dielectric constant of perovskite, ε_0_ is the vacuum permittivity, *e* is the electronic charge, and *L* is the thickness of perovskite.

#### The EIS

EIS measurements were carried out by an EC-lab (SP300).

#### Steady-state PL and TRPL

Steady-state PL and TRPL were measured using a Horiba Fluorolog time-correlated single-photon counting system with photomultiplier tube detectors. The light was illuminated from the top surface of perovskite film. For steady-state PL measurements, the excitation source is a monochromated Xe lamp (peak wavelength at 540 nm with a line width of 2 nm). For TRPL, we used a green laser diode (λ = 504 nm) as the excitation source with an excitation power density of 5 mW/cm^2^. The PL decay curves were fitted with single exponential components to obtain a fast and a slow decay lifetime.

#### Photovoltaic performance characterization

The current density-voltage (*J*-*V*) measurements were performed on a Keithley model 2400 digital source meter controlled by Test point software under a xenon lamp (450 W Xenon, AAA class). The light intensity was calibrated with a National Renewable Energy Laboratory (NREL)-certified KG5-filtered Si reference diode. The areas of all devices are determined by the metal aperture mask atop the devices. For F-PSCs, the aperture area is 0.1486 cm^2^. *J*-*V* curves were obtained by reverse scanning (RS, 1.3 to −0.1 V) and forward scanning (−0.1 to 1.3 V). The voltage step was 0.01 V, and the scanning rate was 0.1 V/s. For 30 cm–by–30 cm modules, the aperture area is 600 cm^2^. *J*-*V* curves were obtained by RS (47.0 to −0.1 V). The voltage step was 0.39 V, and the scanning rate was 3.9 V/s. The areas of the two masks are obtained through the National Photovoltaic Industry Metrology Testing Center certification.
