# Supplementary material for: SnO2 ink engineering for printing efficient flexible perovskite solar modules
Source: Sci Adv. 2025 Oct 24;11(43):eadu1116. doi: 10.1126/sciadv.adu1116 (PMC12551692; doi:10.1126/sciadv.adu1116)
Supplement: Supplementary file 1 — Figs. S1 to S35 Tables S1 to S6 [file sciadv.adu1116_sm.pdf]

Science Advances
AAAS

Supplementary Materials for

SnO_2 ink engineering for printing efficient flexible perovskite solar modules

Chao Wang *et al.*

Corresponding author: Tongle Bu, tongle.bu@whut.edu.cn; Qi Li, liqi1@xhlab.cn;
Fuzhi Huang, fuzhi.huang@whut.edu.cn

Sci. Adv. **11**, eadu1116 (2025)
DOI: 10.1126/sciadv.adu1116

This PDF file includes:

Figs. S1 to S35
Tables S1 to S6

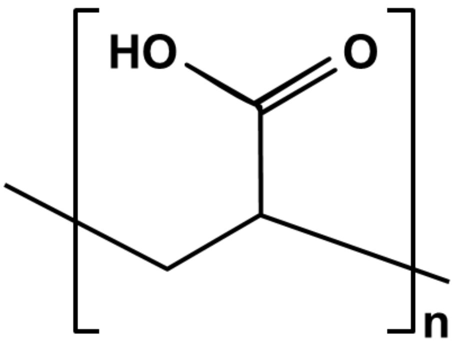

Fig. S1. The molecular formula of PAA. The value of n determines the PAA chain length.

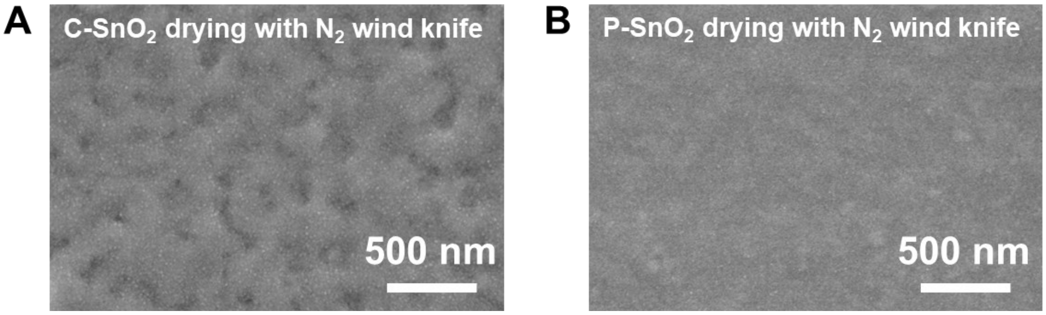

Fig. S2. SEM morphology of SnO_2 films with air knife assistance. SEM morphology of (A) C-SnO_2 and (B) P-SnO_2 films drying with N_2 wind knife.

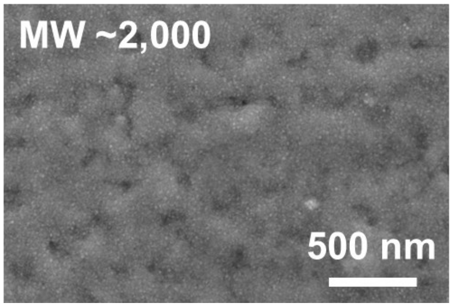

Fig. S3. SEM morphology of P-SnO_2 film modified with low molecular weight (MW) PAA. SEM morphology of SnO_2 film with PAA (MW ~2,000).

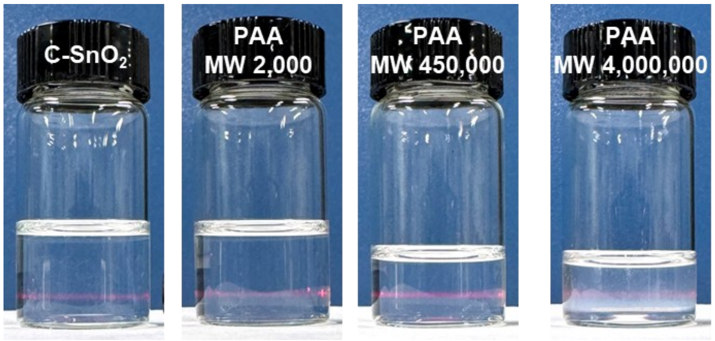

Fig. S4. Digital images of different SnO_2 inks. Digital images of C-SnO_2 ink and P-SnO_2 inks with various PAA MWs.

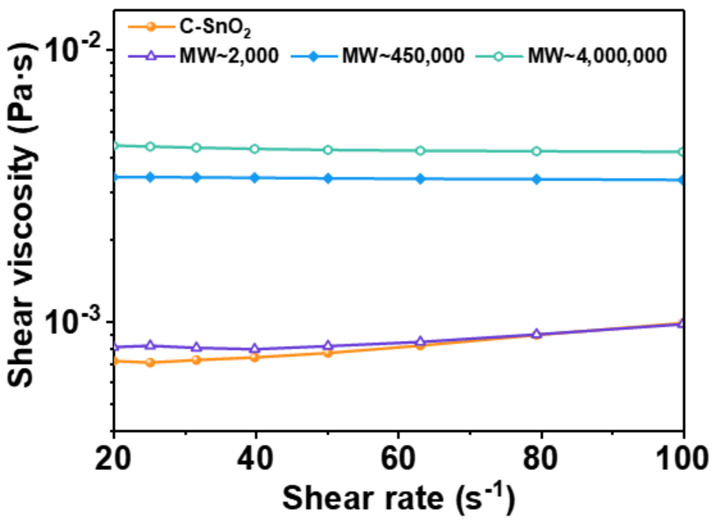

Fig. S5. Shear viscosity measurement of different SnO_2 inks. The shear viscosity-shear rates curves of C-SnO_2 ink and P-SnO_2 inks with different molecular weights (MW) of PAA.

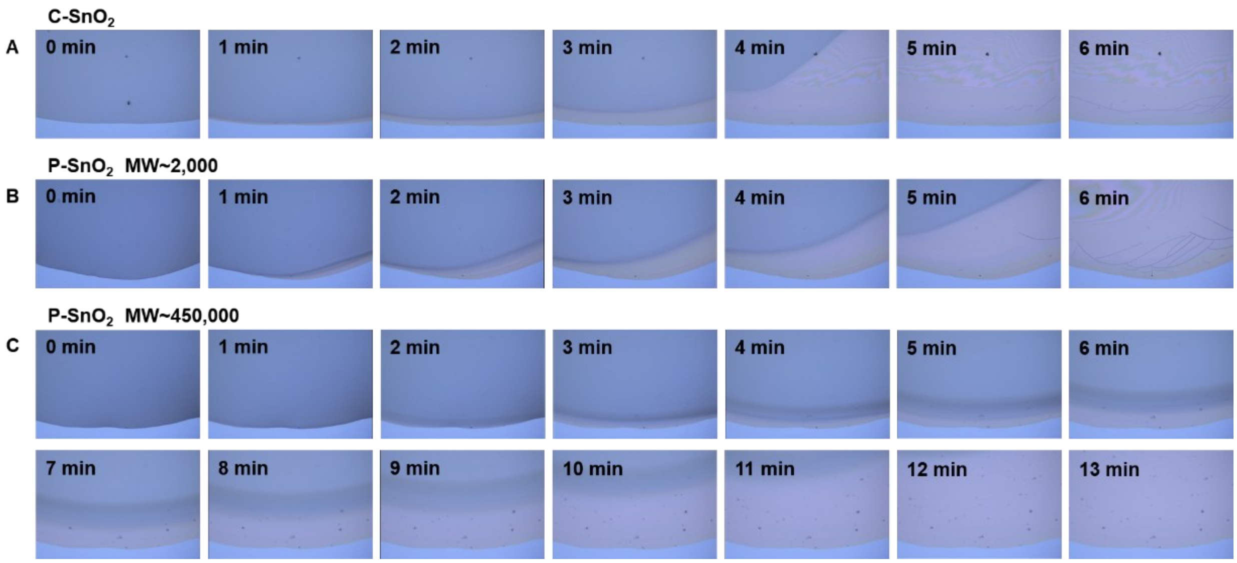

Fig. S6. Monitoring of the evaporation process of different SnO_2 ink droplets. Microscope images of (A) C-SnO_2 inks droplets, and P-SnO_2 inks droplets with PAA of (B) MW of ~2,000 and (C) MW~450,000 over time under natural drying conditions without airflow interference.

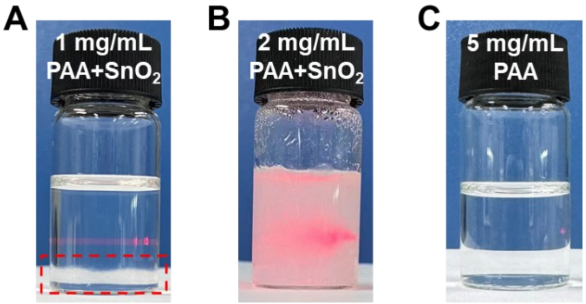

Fig. S7. Digital images of P-SnO_2 inks with varying PAA concentrations and PAA aqueous solution. Digital images of P-SnO_2 inks with (A) 1 mg/mL and (B) 2 mg/mL PAA, and (C) 5 mg/mL PAA aqueous solution without SnO_2 NCs.

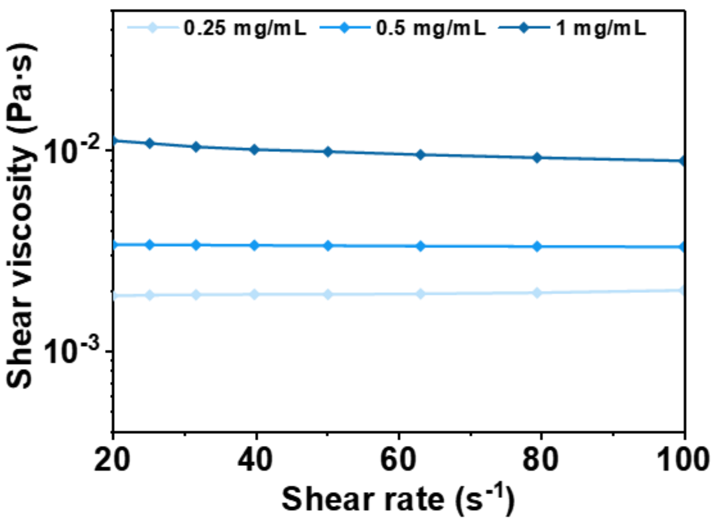

Fig. S8. Shear viscosity measurement of P-SnO_2 inks with different concentrations of PAA. The shear viscosity-shear rates curves of P-SnO_2 inks with different concentrations of PAA.

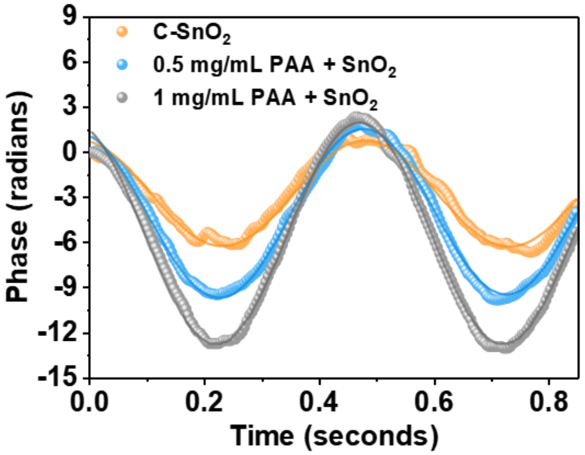

Fig. S9. The Zeta potential measurement of different SnO_2 inks. The Zeta potential curves of different SnO_2 ink.

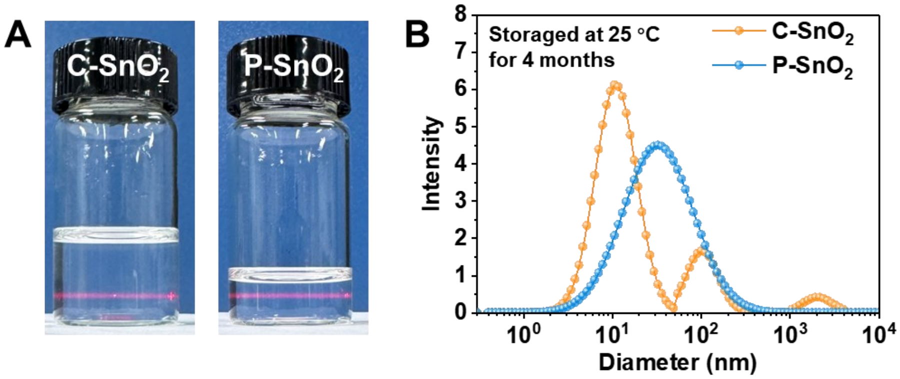

Fig. S10. The storage stability of different SnO_2 inks. (A) Digital photos of SnO_2 inks after 4 months of storage. (B) DLS curves of SnO_2 inks after 4 months of storage.

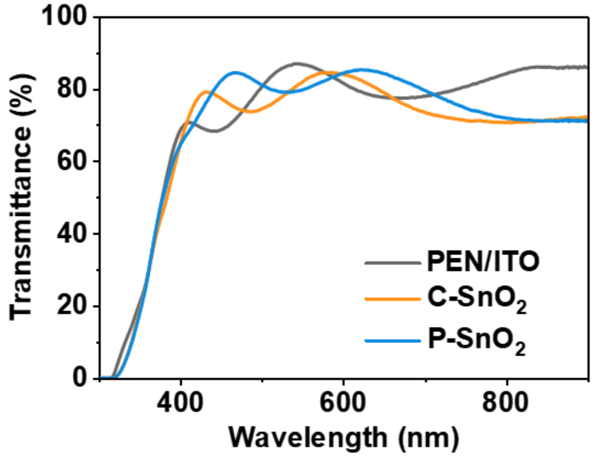

Fig. S11. UV-vis transmittance spectra of flexible substrate and different SnO_2 films on flexible substrate. The transmittance spectra of PEN/ITO substrate, and C-SnO_2 and P-SnO_2 films on PEN/ITO substrate.

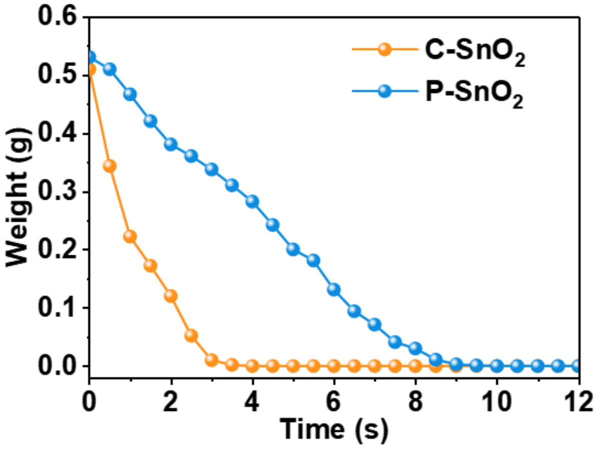

Fig. S12. Weight monitoring of the slot-die coating wet SnO_2 film self-volatilization process. The weight of C-SnO_2 and P-SnO_2 wet films by slot-die coating over time.

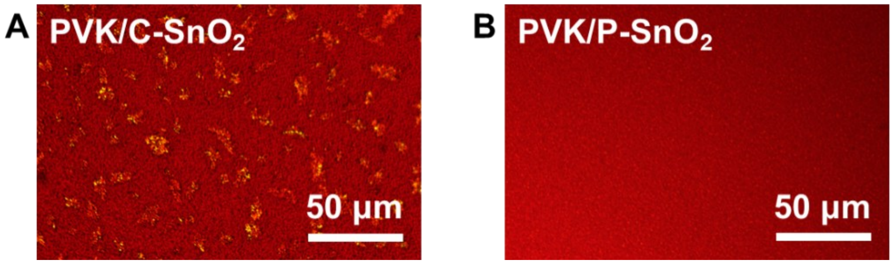

Fig. S13. Optical microscope images of PVK films. The transmission optical microscope images of PVK films deposited on (A) C-SnO_2 and (B) P-SnO_2 films.

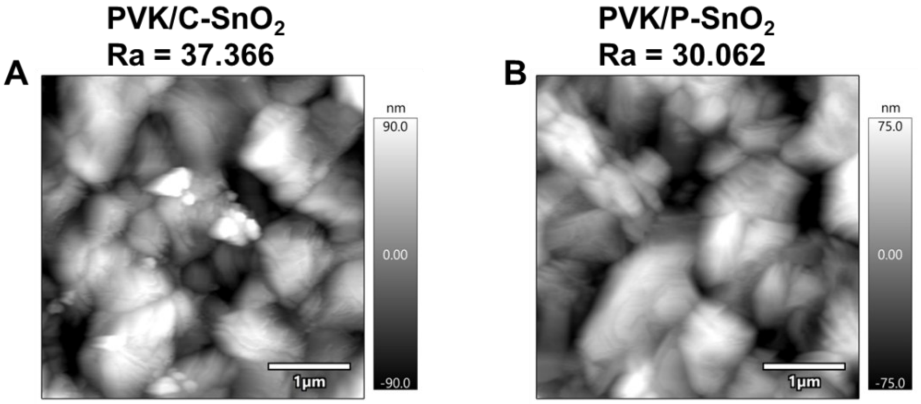

Fig. S14. AFM morphology of PVK films. AFM morphology of PVK films on (A) C-SnO_2 and (B) P-SnO_2.

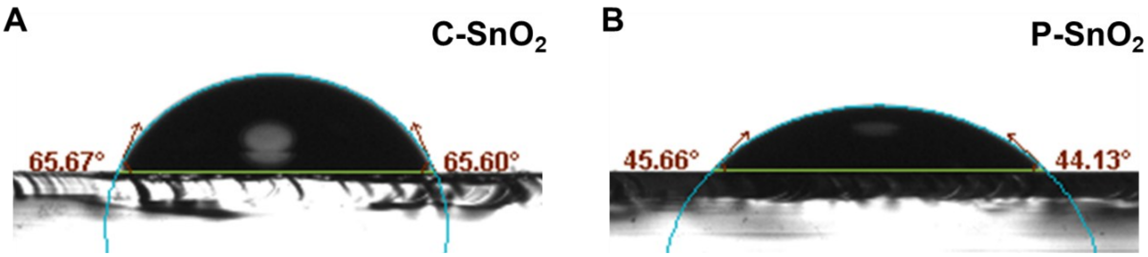

Fig. S15. Contact angle images of PVK precursor solution on different SnO_2 films surfaces. Contact angle images of PVK precursor solution dropped on (A) C-SnO_2 and (B) P-SnO_2.

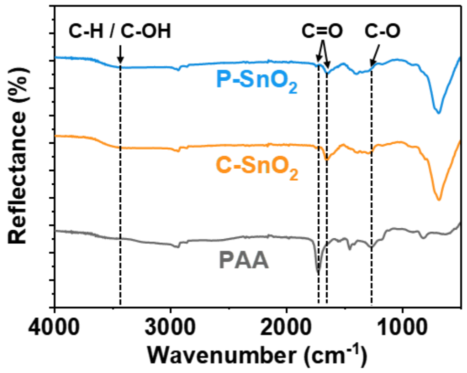

Fig. S16. The total reflection mode FTIR curves. The total reflection mode FTIR curves of PAA, C-SnO_2 and PAA-SnO_2 deposited on ITO glass substrate.

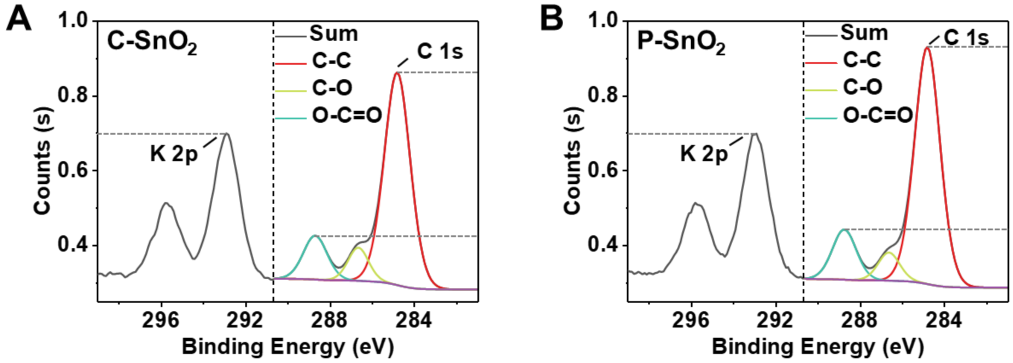

Fig. S17. XPS characterization of different SnO_2. XPS spectrum of C 1s and K 2p for (A) C-SnO_2 and (B) P-SnO_2 films.

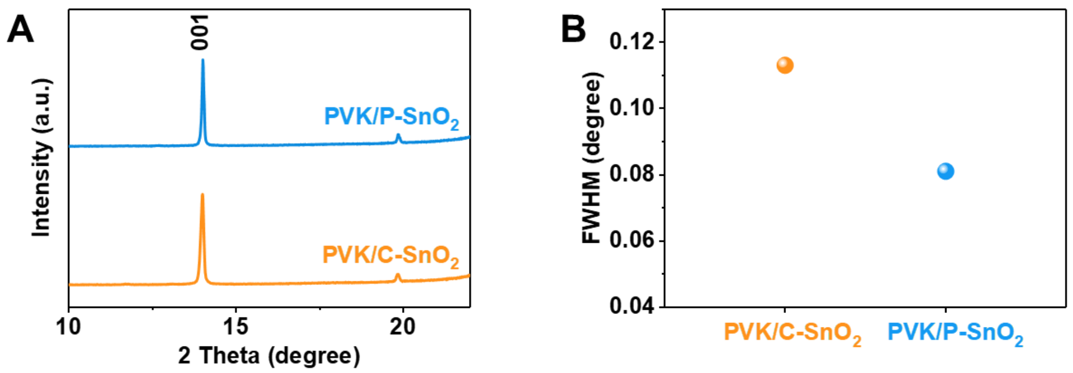

Fig. S18. XRD patterns of perovskite films deposited on different SnO_2. (A) XRD curves and (B) FWHM value of PVK deposited on C-SnO_2 and P-SnO_2 films.

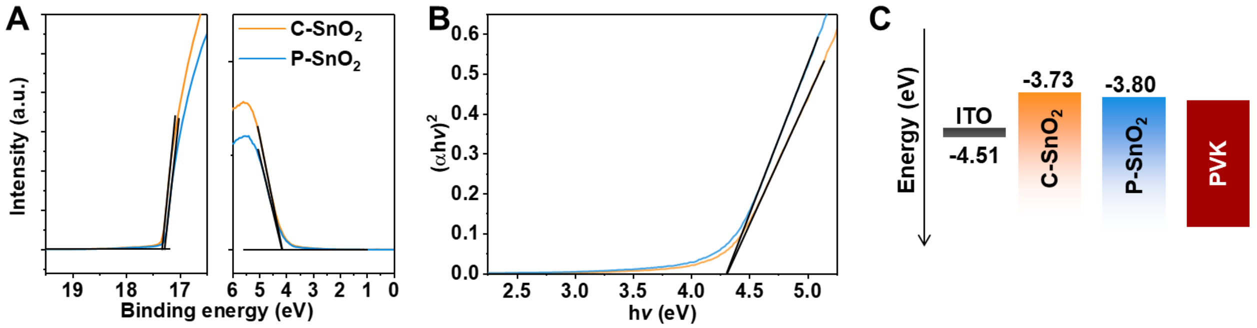

Fig. S19. Energy level measurement via UPS and UV-vis characterization. (A) UPS and (B) UV-vis curves of C-SnO_2 and P-SnO_2 films. (C) Diagram of energy level structure of PVK, C-SnO_2, and P-SnO_2.

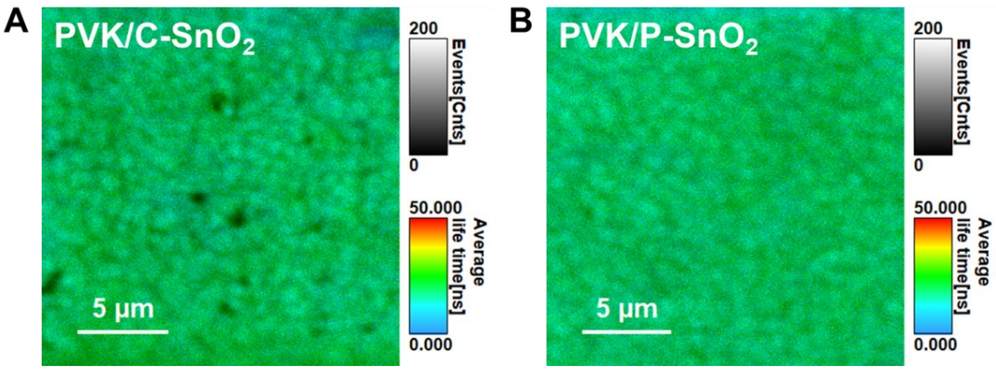

Fig. S20. Time-resolved confocal photoluminescence mappings of PVK films deposited on different SnO_2. Time-resolved confocal photoluminescence mappings of PVK films on the (A) C-SnO_2 and (B) P-SnO_2 ETLs. Note that the average carrier lifetime data (color scale bars) and the event count data (grey scale bars) have been merged.

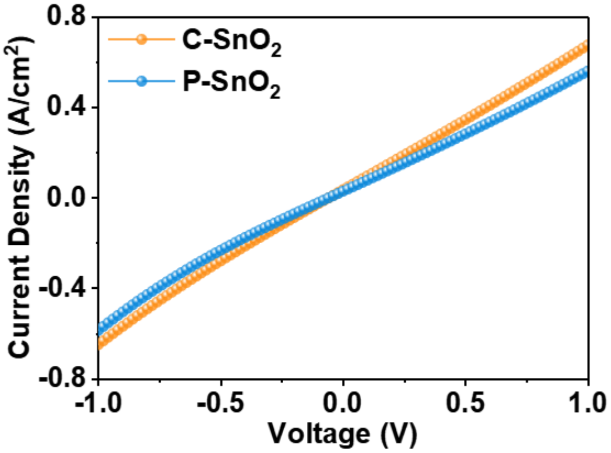

Fig. S21. Conductivity measurement of different SnO_2 films. Conductivity curves of C-SnO_2 and P-SnO_2 films by the structure of PEN/ITO/SnO_2/Au.

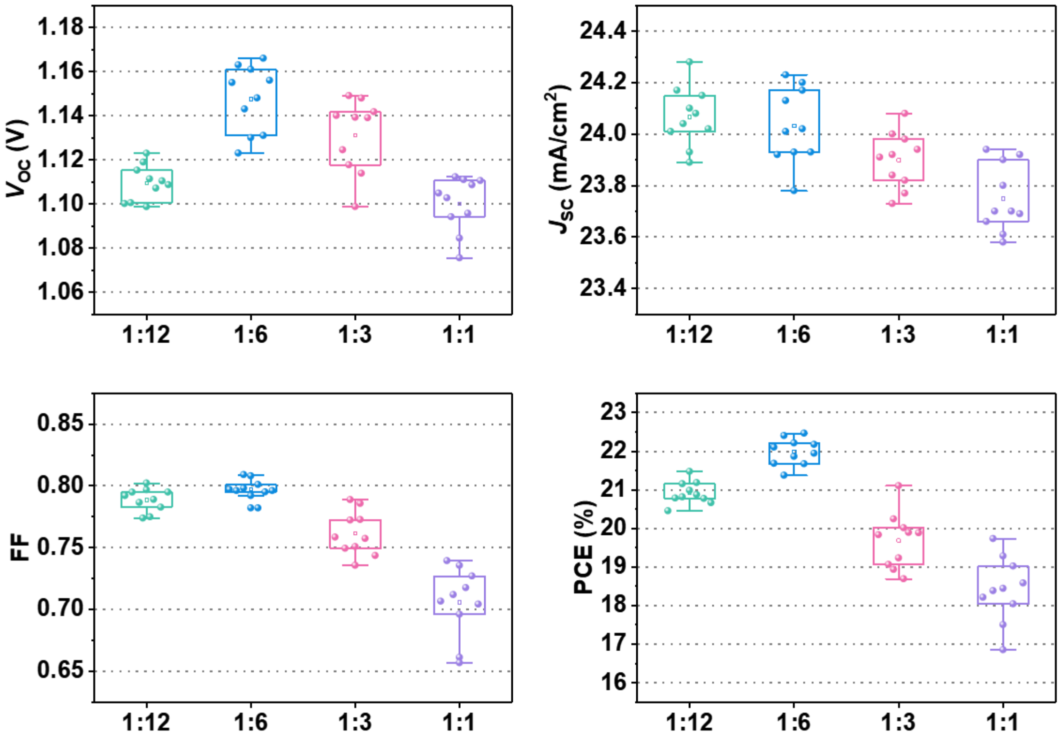

Fig. S22. The correlation between SnO_2 thickness and device performance. The SnO_2 layer thickness was controlled by varying the volume ratio (volume of SnO_2 NCs : total volume) to 1 : 12, 1 : 6, 1 : 3, and 1 : 1, respectively. Device counts are 10 for each type. The top and bottom horizontal lines of the box plots denote the 75th percentile and the 25th percentile, respectively. The middle point represents the average value. The whiskers correspond to an outlier detection coefficient of 1.5.

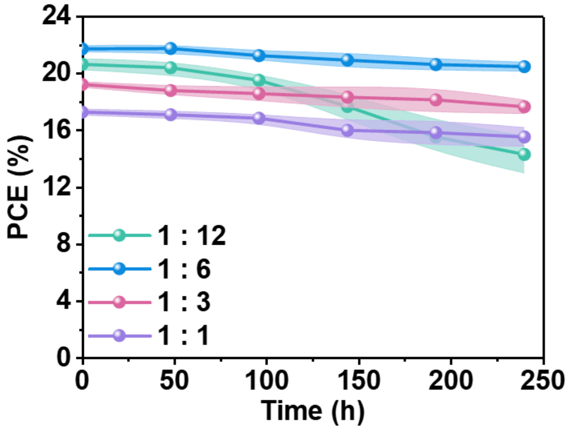

Fig. S23. Illumination stability characterization of flexible PSCs based on different thickness P-SnO_2. Light aging test of the unencapsulated flexible PSCs exposed under a white LED light in the N_2 environment (25 ± 5 °C). Five unencapsulated flexible PSCs for each type. Data are presented as mean values ± standard deviation. After 240 hours of illumination, the average efficiency of these four samples with low to high thickness decreased to 69.2%, 94.2%, 93.9%, and 89.9% of their initial values, respectively.

中国科学院电工研究所太阳光伏发电系统和风力发电系统质量检测中心

报告编号:PWQC-WT-P23051621-1R

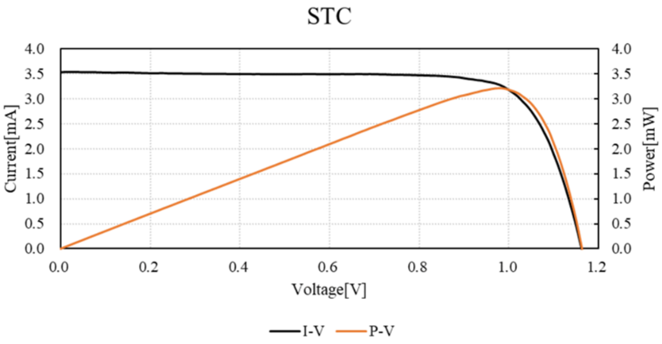

**Type**	单结柔性钙钛矿太阳电池
**Ser.No**	DC2023a038
**Area**	0.14860 cm^2
**Isc**	3.522 mA
**Jsc**	23.704 mA/cm^2
**Voc**	1.162 V
**FF**	78.24 %
**Pm**	3.203 mW
**E$_{ff}$**	21.56 %
**Ipm**	3.269 mA
**Vpm**	0.980 V
**Voltage Sweep**	Reverse
**Sweep time**	1.31 s
**Temp**	25 ℃
**Irr**	100 mW/cm^2
**File**	A20230516093514

Fig. S24. Small-sized devices certification report. The efficiency certified report tested in the institute of Electrical Engineering of the Chinese Academy of Sciences. The *J-V* curve of the certified P-SnO_2 based F-PSC with a PCE of 21.56% under reverse scan on an aperture area of 0.1486 cm^2.

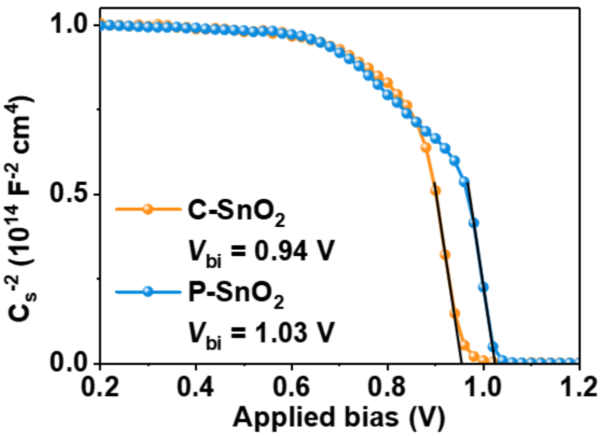

Fig. S25. Mott-Schottky curves of the F-PSCs based on different SnO_2 films. Mott-Schottky curves of the F-PSCs based on (A) C-SnO_2 and (B) P-SnO_2 films.

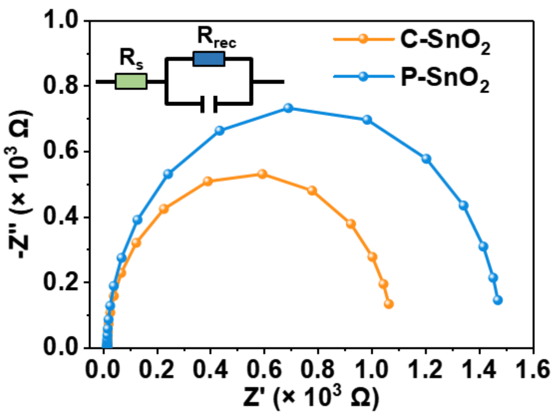

Fig. S26. EIS curves of the F-PSCs based on different SnO_2 films. EIS curves of the F-PSCs based on (A) C-SnO_2 and (B) P-SnO_2 films.

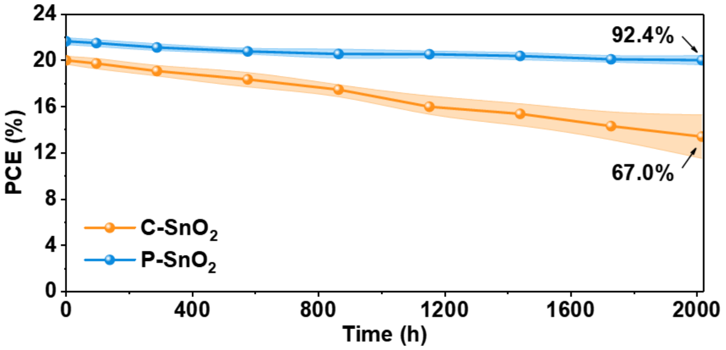

Fig. S27. Storage stability of unencapsulated small-size devices. Storage stability of unencapsulated F-PSCs (0.1486 cm^2) under the ambient condition (~20% RH, ~25 °C).

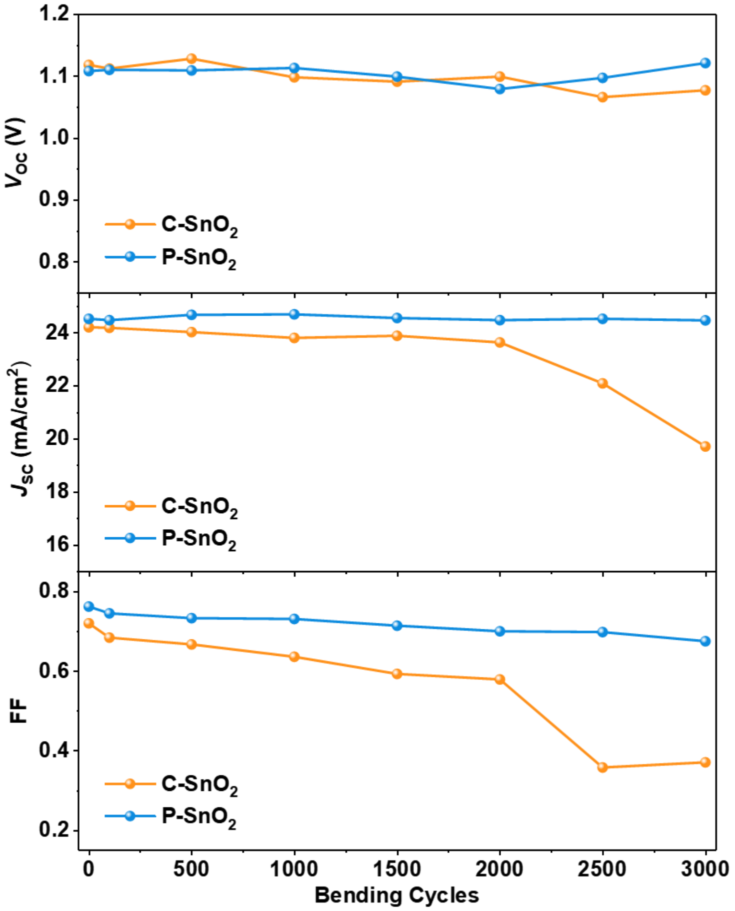

Fig. S28. Bending durability of unencapsulated small-size devices. Bending durability of unencapsulated F-PSCs with bending cycles (R = 5 mm, ~1 Hz, tension-only, in ambient [~20% RH, ~25 °C]).

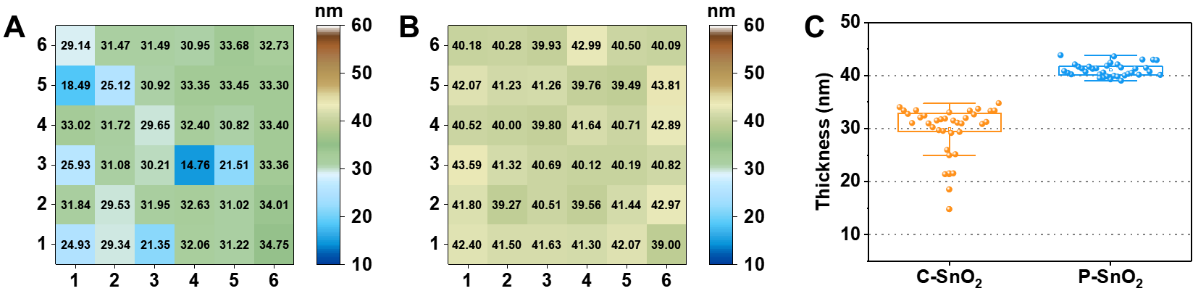

Fig. S29. Thickness distribution of different 30 cm × 30 cm SnO_2 films. Color heatmaps of ellipsometry thickness for (A) C-SnO_2 and (B) P-SnO_2 films. 36 samples are evenly divided from 30 cm × 30 cm SnO_2 films. (C) The thickness distribution of different SnO_2 films.

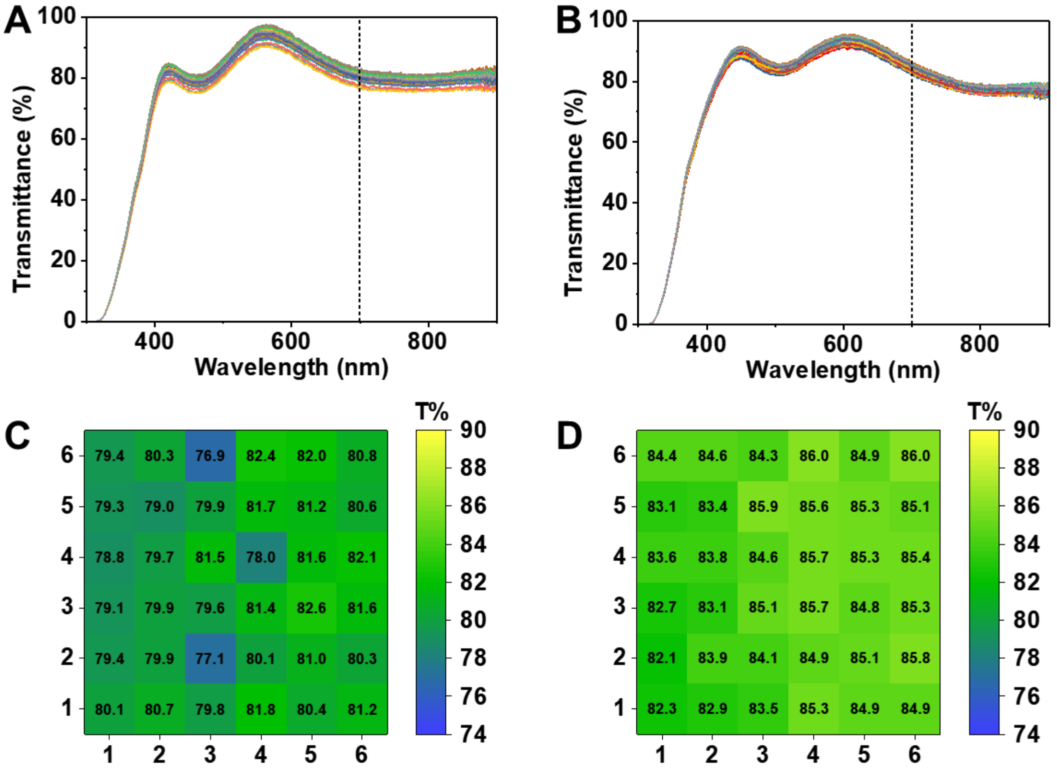

Fig. S30. Transmittance distribution of different 30 cm × 30 cm SnO_2 films. Transmittance curves of (A) C-SnO_2 and (B) P-SnO_2 films. Color heatmaps of transmittance (at the wavelength of 700 nm) for (C) C-SnO_2 and (D) P-SnO_2 films. The 36 samples were evenly divided from 30 cm × 30 cm SnO_2 films.

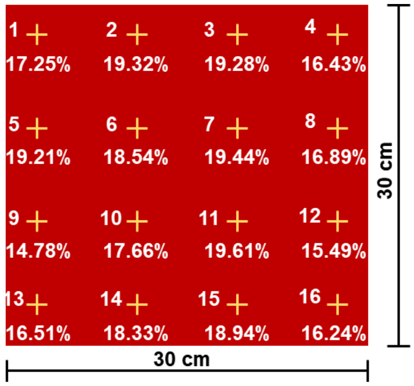

Fig. S31. Efficiency distribution of 30 cm × 30 cm devices. C-SnO_2 Efficiency distribution of small-area C-SnO_2 based F-PSCs cut from 30 cm × 30 cm films.

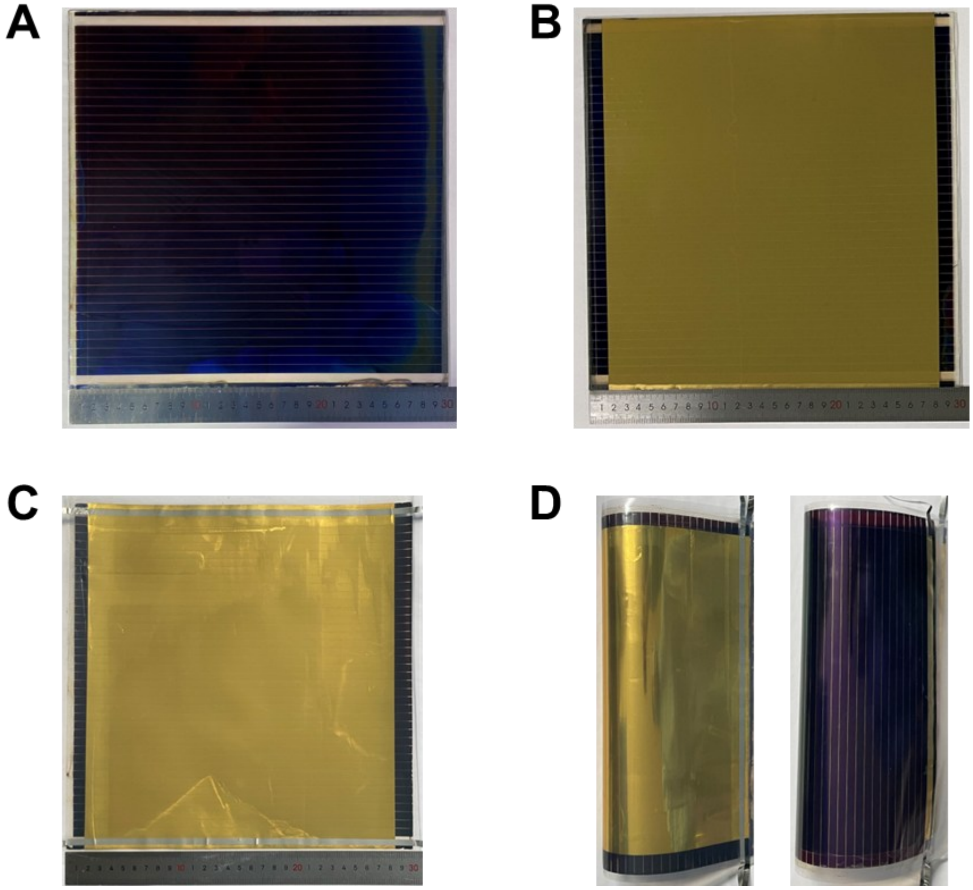

Fig. S32. Digital photos of 30 cm × 30 cm F-PSM. (A) The PVK/Spiro-OMeTAD film deposited by slot-die on 30 cm × 30 cm PEN/ITO/P-SnO_2. (B) The 30 cm × 30 cm F-PSM. (C) The encapsuled 30 cm × 30 cm F-PSM. (D) The bended 30 cm × 30 cm F-PSM.

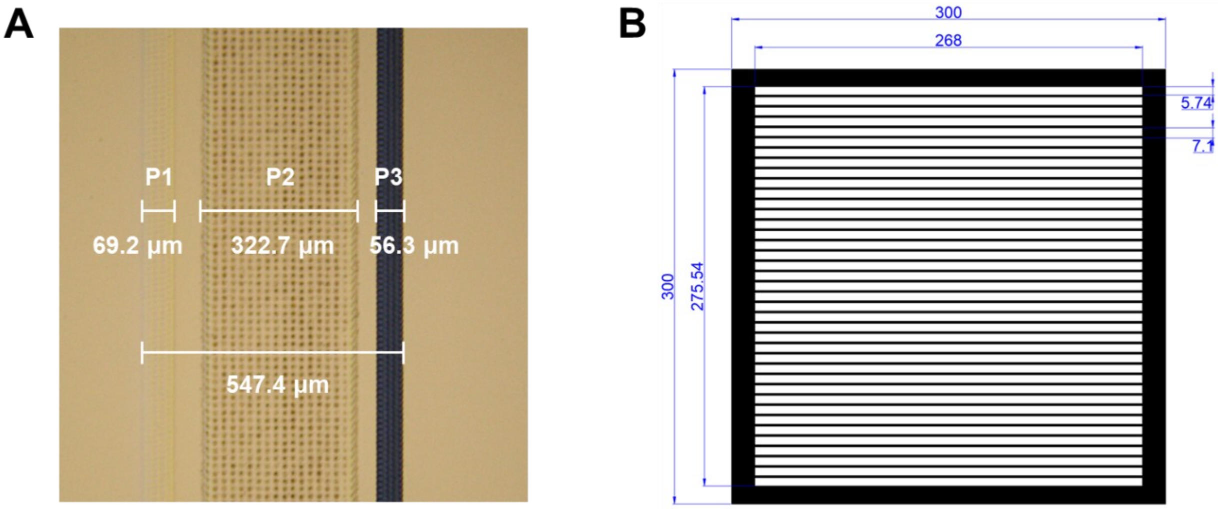

Fig. S33. Design of the 30 cm × 30 cm flexible sub-module. (A) The optical microscopy image of P1, P2 and P3 scribing lines. (B) The mask drawing with a mask area of 600 cm^2, in which all dimensions are in millimeters.

Report No.: B23X91983 Page 2 of 3

1	TABLE: Visual inspection		—
Test Date [MM/DD/YYYY]..................:	07/25/2023		—
Sample No.	Nature and position of initial findings – comments or attach photos		Verdict
712B	No major visual defects		—
Supplementary information: Module active area is 0.06 [m²], module size is 300 mm * 300 mm			

2	TABLE: Maximum power determination						—
Test Date [MM/DD/YYYY]..................:	07/25/2023						—
Radiant Source................................:	☒ Solar Simulator ☐ Natural Sunlight						—
Irradiance [W/m²] ....:	1000						—
Sample No.	Voc [V]	Vmp [V]	Isc [A]	Imp [A]	Pmp [W]	FF [%]	Power conversion efficiency (%)
712B	39.33	30.25	0.38	0.32	9.77	66.57	16.28

Supplementary information:

Power conversion efficiency: the efficiency calculated based on the designated illumination area, which is a portion of the cell or module area from which some cell or module contacting components are excluded. The designated illumination area of the sample in this report is provided by the applicant, and the designated illumination area of the test sample in this report is 600 cm^2.

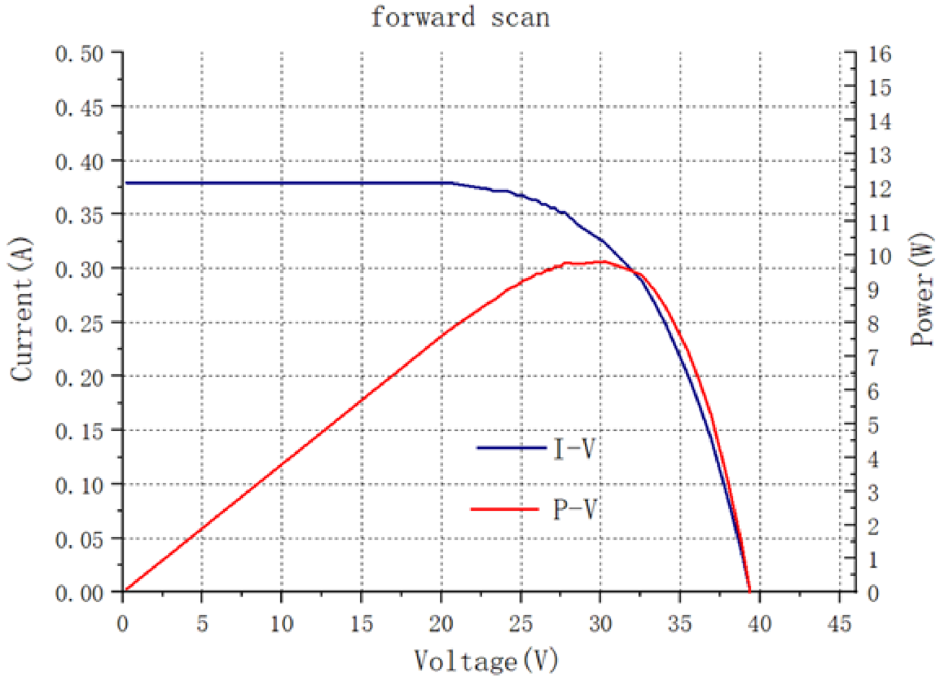

Fig. S34. Sub-modules certification report. Efficiency certified report tested in China Academy of Information and Communications Technology (CAICT). The *I-V* curve of the certified P-SnO_2 based F-PSM with a PCE of 16.28% under reverse scan on an aperture area of 600 cm^2.

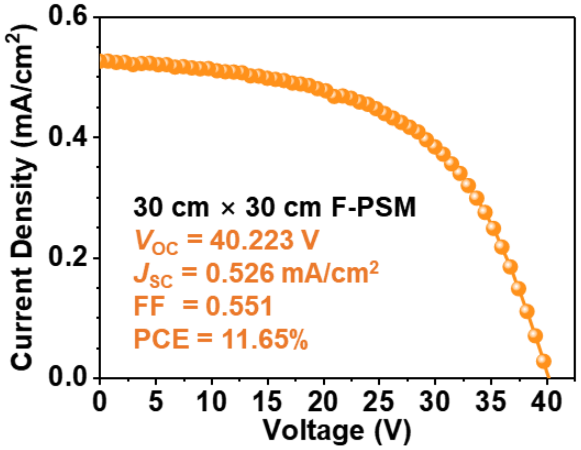

Fig. S35. The *J-V* curve in RS of C-SnO_2 based F-PSM. The *J-V* curve in RS of C-SnO_2 based F-PSM with aperture area of 600 cm^2.

Table S1. The fitting parameters of Zeta potential of different SnO_2 ink.

Colloidal solutions	Zeta potential (mV)
C-SnO_2	−24.82
0.5 mg/mL PAA + SnO_2	−40.70
1 mg/mL PAA + SnO_2	−49.07

Table S2. The XPS peak intensity of K 2p and C 1s.

Samples	K 2p	C 1s		
		O-C=O	C-O	C-C
C-SnO_2	697.6	425.6	392.7	862.9
P-SnO_2	696.2	442.7	379.8	927.4

Table S3. The parameters calculated from UPS and UV-vis.

Samples	E_g	$E_{cut-off}$	E_{onset}	W_F	VBM	CBM
C-SnO_2	4.31	17.33	4.15	3.89	-8.04	-3.73
P-SnO_2	4.31	17.29	4.18	3.93	-8.11	-3.80

Table S4. The fitting parameters of TRPL spectra by single exponential fitting.

	PVK/C-SnO_2 (μs)	PVK/P-SnO_2 (μs)
A	0.798	0.810
τ	0.641	0.393

Table S5. The parameters of the best-performing F-PSCs based on C-SnO_2 and P-SnO_2 ETLs.

Samples	Scan	V_{OC} (V)	J_{SC} (mA/cm^2)	FF	PCE (%)
C-SnO_2	RS	1.120	23.93	0.786	21.07
	FS	1.087	23.95	0.662	17.23
P-SnO_2	RS	1.166	24.23	0.795	22.46
	FS	1.163	24.30	0.797	22.54

Table S6. The *J-V* parameters in RS of the F-PSCs based on 16 samples of C-SnO_2 and P-SnO_2.

Samples	Number	V_{OC} (V)	J_{SC} (mA/cm^2)	Fill Factor	PCE (%)
C-SnO_2	1	1.071	22.37	0.720	17.25
	2	1.120	21.84	0.790	19.32
	3	1.120	22.06	0.780	19.28
	4	1.121	23.05	0.636	16.43
	5	1.095	23.87	0.735	19.21
	6	1.116	23.71	0.701	18.54
	7	1.129	22.58	0.762	19.44
	8	1.033	23.63	0.692	16.89
	9	1.002	23.41	0.630	14.78
	10	1.092	23.44	0.690	17.66
	11	1.106	23.37	0.759	19.61
	12	0.994	21.11	0.738	15.49
	13	0.995	23.03	0.721	16.51
	14	1.067	23.40	0.734	18.33
	15	1.085	23.21	0.752	18.94
	16	1.036	20.82	0.753	16.24
P-SnO_2	1	1.092	24.74	0.771	20.84
	2	1.100	23.63	0.810	21.08
	3	1.100	23.58	0.812	21.05
	4	1.106	23.69	0.814	21.32
	5	1.106	23.68	0.813	21.28
	6	1.108	23.70	0.802	21.06
	7	1.104	23.64	0.806	21.02
	8	1.105	23.72	0.805	21.09
	9	1.098	23.73	0.799	20.83
	10	1.128	23.69	0.790	21.11
	11	1.132	23.94	0.769	20.85
	12	1.129	23.70	0.799	21.39
	13	1.130	23.86	0.779	20.99
	14	1.138	23.83	0.789	21.38
	15	1.118	23.66	0.806	21.33
	16	1.124	23.71	0.793	21.12
